# Publisher Correction: The role of calcium in regulating marine phosphorus burial and atmospheric oxygenation

**DOI:** 10.1038/s41467-020-16793-6

**Published:** 2020-06-11

**Authors:** Mingyu Zhao, Shuang Zhang, Lidya G. Tarhan, Christopher T. Reinhard, Noah Planavsky

**Affiliations:** 1 Department of Geology and Geophysics, Yale University, 210 Whitney Ave, New Haven, CT 06511 USA; 2 School of Earth and Atmospheric Sciences, Georgia Institute of Technology, Atlanta, GA 30332 USA

**Keywords:** Carbon cycle, Element cycles, Palaeoceanography, Marine chemistry

Correction to: *Nature Communications* 10.1038/s41467-020-15673-3, published online 06 May 2020.

The original version of the Supplementary Information associated with this Article omitted Supplementary Data 2, 3, and 4 which contained the code used in this study. The HTML has been updated to include Supplementary Data 2, 3, and 4.

