# Supplementary material for: The role of calcium in regulating marine phosphorus burial and atmospheric oxygenation
Source: Nat Commun. 2020 May 6;11:2232. doi: 10.1038/s41467-020-15673-3 (PMC7203231; doi:10.1038/s41467-020-15673-3)
Supplement: Supplementary file 1 — Supplementary Information [file 41467_2020_15673_MOESM1_ESM.pdf]

Supplementary Information

The role of calcium in regulating marine phosphorus burial and atmospheric oxygenation

Zhao et al.

Nature Communications

Supplementary Methods

1. The 'Extended' 1D Multicomponent Diagenetic Model: SEDCHEM

Derivation of pH formulation

If the dissociation constants do not change with time, TP can be written as a function of proton concentration and the total concentrations of dissociation systems, which is

$$TP = f(H, [S_j]) \tag{S1}$$

Thus, the time derivative of the total proton balance can be written as

$$\frac{dTP}{dt} = \frac{\partial TP}{\partial H}\frac{dH}{dt} + \sum_j \frac{\partial TP}{\partial [S_j]}\frac{d[S_j]}{dt} \tag{S2}$$

Rearranging the equation gives

$$\frac{dH}{dt} = (\frac{dTP}{dt} - \sum_j \frac{\partial TP}{\partial [S_j]}\frac{d[S_j]}{dt})/\frac{\partial TP}{\partial H} \tag{S3}$$

Substituting $\mathrm{CO_2} = \frac{\mathrm{H^2}}{\mathrm{H^2+K_1H+K_1K_2}}\sum \mathrm{CO_2}$, $\mathrm{HCO_3^-} = \frac{\mathrm{K_1H}}{\mathrm{H^2+K_1H+K_1K_2}}\sum \mathrm{CO_2}$, $\mathrm{NH_4^+} = \frac{\mathrm{H}}{\mathrm{H+K_N}}\sum \mathrm{NH_3}$, $\mathrm{H_2PO_4^-} = \frac{\mathrm{K_{P1}H^2}}{\mathrm{H^3+K_{P1}H^2+K_{P1}K_{P2}H+K_{P1}K_{P2}K_{P3}}}\sum \mathrm{H_3PO_4}$, $\mathrm{HPO_4^{2-}} = \frac{\mathrm{K_{P1}K_{P2}H}}{\mathrm{H^3+K_{P1}H^2+K_{P1}K_{P2}H+K_{P1}K_{P2}K_{P3}}}\sum \mathrm{H_3PO_4}$ and $\mathrm{H_2S} = \frac{\mathrm{H}}{\mathrm{H+K_{S1}}}\sum \mathrm{H_2S}$ in Equation 10, the formulations of $\frac{\partial TP}{\partial [S_j]}$ and $\frac{\partial TP}{\partial H}$ can then be obtained as

$$\frac{\partial TP}{\partial \sum \mathrm{CO_2}} = 2\frac{\mathrm{H^2}}{\mathrm{H^2+K_1H+K_1K_2}} + \frac{\mathrm{K_1H}}{\mathrm{H^2+K_1H+K_1K_2}} \tag{S4}$$

$$\frac{\partial TP}{\partial \sum \mathrm{NH_3}} = \frac{\mathrm{H}}{\mathrm{H+K_N}} \tag{S5}$$

$$\frac{\partial TP}{\partial \sum H_3PO_4} = 2\frac{K_{P1}H^2}{H^3+K_{P1}H^2+K_{P1}K_{P2}H+K_{P1}K_{P2}K_{P3}} + \frac{K_{P1}K_{P2}H}{H^3+K_{P1}H^2+K_{P1}K_{P2}H+K_{P1}K_{P2}K_{P3}} \quad (S6)$$

$$\frac{\partial TP}{\partial \sum H_2S} = \frac{H}{H+K_{S1}} \quad (S7)$$

$$\frac{\partial TP}{\partial H} = 2\frac{2H(H^2+K_1H+K_1K_2)-(2H+K_1)H^2}{(H^2+K_1H+K_1K_2)^2}\sum CO_2 + \frac{K_1(H^2+K_1H+K_1K_2)-(2H+K_1)K_1H}{(H^2+K_1H+K_1K_2)^2}\sum CO_2 + \frac{K_N}{(H+K_N)^2}\sum NH_3 + 2\frac{2K_{P1}H(H^3+K_{P1}H^2+K_{P1}K_{P2}H+K_{P1}K_{P2}K_{P3})-(3H^2+2K_{P1}H+K_{P1}K_{P2})K_{P1}H^2}{(H^3+K_{P1}H^2+K_{P1}K_{P2}H+K_{P1}K_{P2}K_{P3})^2}\sum H_3PO_4 + \frac{K_{P1}K_{P2}(H^3+K_{P1}H^2+K_{P1}K_{P2}H+K_{P1}K_{P2}K_{P3})-(3H^2+2K_{P1}H+K_{P1}K_{P2})K_{P1}K_{P2}H}{(H^3+K_{P1}H^2+K_{P1}K_{P2}H+K_{P1}K_{P2}K_{P3})^2}\sum H_3PO_4 + \frac{K_{S1}}{(H+K_{S1})^2}\sum H_2S \quad (S8)$$

Derivation of Adsorption formulation

The total iron (T_{Fe}) in the modeled system is defined as the sum of Fe^{2+} and A_{Fe}. If calibrated to the volume of solute, the concentration of total iron is

$$T_{Fe} = Fe^{2+} + FA_{Fe} = (1 + K_{Fe})Fe^{2+} \quad (S9)$$

The derivative of T_{Fe} is simply the sum of the reaction and transport terms of Fe^{2+} and A_{Fe}, which is

$$\frac{dT_{Fe}}{dt} = FRT_{A_{Fe}} + RT_{Fe^{2+}} \quad (S10)$$

Combining Equations S9 and S10 yields

$$\frac{dFe^{2+}}{dt} = \frac{F}{1+K_{Fe}}RT_{A_{Fe}} + \frac{1}{1+K_{Fe}}RT_{Fe^{2+}} \quad (S11)$$

The adsorption process also involves the transfer of protons. For example, the equation for the adsorption of Fe^{2+} can be written as

$$A_H + Fe^{2+} \rightarrow A_{Fe} + H^+ \quad (S12)$$

where A_H is the adsorbed proton. The derivative of Fe^{2+} can also be written as

$$\frac{dFe^{2+}}{dt} = RT_{Fe^{2+}} - RA_H \quad (S13)$$

Combining Equations S11 and S13 yields

$$RA_H = \frac{K_{Fe}}{1+K_{Fe}} RT_{Fe^{2+}} - \frac{F}{1+K_{Fe}} RT_{A_{Fe}} \quad (S14)$$

Model Solution

We employed model domain thicknesses of 300 cm for initial model runs and comparison to empirical data from the "Friends of Anoxic Mud" (FOAM) site in Long Island Sound[1-9]. The model was developed in R[10]. The R package ReacTran was used to simplify the transport codes[11]. The model was solved by the Method of Lines using the Variable Coefficient Ordinary Differential Equations (VODE) solver[12]. The model was run dynamically to steady state, although the upper 15 cm of most marine sediments, including FOAM, are likely subject to strong seasonal variations[2].

Model Application

We selected FOAM to evaluate the robustness of our model given that it is a site from which empirical data on bioturbation intensities and sediment mixed layer depths have been collected, various porewater chemical species and complete P speciation such as iron-bound P, authigenic P and organic P have been measured, a complete redox balance has been determined, and various diagenetic processes have been extensively investigated[1-9]. Collection of this entire suite of sediment biogeochemical and bioturbation data from a single site is surprisingly rare and is an obvious target for future work to explore the robustness of this model. The majority of reaction rate constants employed in the model are adopted from previous studies (Table S5), with the exception of those for calcite, aragonite and CFA precipitation and biotite dissolution, which were adjusted to reproduce the FOAM geochemical profiles. However, it is important to note that these adjusted rates

are not anomalous relative to those expected in continental margin sediments. Authigenic P phases measured at FOAM by the SEDEX method include CFA, biogenic apatite, $CaCO_3$ and smectite[13]. While CFA formed primarily from porewater, the other authigenic P phases may actually be detrital or have precipitated from seawater. To reproduce the authigenic P profile of the FOAM site, we also applied an authigenic P flux term from seawater to sediments (e.g., biogenic apatite, $CaCO_3$ and/or smectite). The precipitation of CFA from seawater is likely hindered by undersaturation of precursor phases such as octacalcium phosphate[14]. Vivianite has not been found at the FOAM site, due to low Fe concentrations through most of the sediment pile.

Model Results for the FOAM Site

Our 'extended' diagenetic model can reproduce multiple sedimentary geochemical profiles recorded from FOAM (Fig. S2), including Mn^{2+}, Fe^{2+}, sulfate, methane, magnetite, ammonia, Ca^{2+}, DIC, phosphate, and other P species. Our model also reproduces measured pH. As most of the diagenetic reactions employed in the model (including calculation of CFA saturation state) involve proton exchange and thus influence porewater pH (Table S2), realistic parameterization of pH is an essential criterion; the ability of our model to accurately calculate pH therefore suggests that our treatment of CFA formation and P cycling more generally are robust.

Our model can also accurately reproduce FOAM organic P profiles (Fig. S2) and regeneration rates. It is likely that the remineralization of organic P is not enough to sharply increase the dissolved phosphate concentration near the sediment-seawater interface, as is observed at FOAM (Fig. S2). Thus, some other phosphate source such as iron-bound P is required[8], which is further supported by the substantial decrease in inorganic P observed in the top 10 cm at FOAM[2]. As the iron-bound P measured at FOAM by the SEDEX method is below detection limits[8], it is possible that most of the iron-bound P has been released to porewaters in the uppermost portion of the sediment pile (Fig. S2, ref. 2). There is also some uncertainty regarding the magnitude of the flux of authigenic P from seawater to the sediment pile at FOAM[8], which may be biogenic apatite and/or P associated with smectite. It is challenging to infer the seawater-to-sediment authigenic P flux solely from solid-phase authigenic P data, due to the paucity of data collected from the immediate

vicinity of the sediment-seawater interface and uncertainty regarding the influence of biodiffusion at this interface. In our 'best-fit' model result, this authigenic P flux is 5.27×10^{-5} mmol cm^{-2} yr^{-1}, equivalent to a concentration of 1.41 μmol g^{-1} within the sediment column.

The total solid-phase P flux (organic P, iron-bound P and authigenic P) from seawater to sediment is, in our 'best-fit' model output, 0.0037 mmol cm^{-2} yr^{-1}, while the dissolved phosphate flux from sediment to seawater is 0.0024 mmol cm^{-2} yr^{-1}. This latter value is very similar to the fall (seasonal) dissolved phosphate flux of 0.0021 mmol cm^{-2} yr^{-1} directly measured by Aller[15], which falls between the values of the empirically observed summer and winter fluxes. The model results show that P burial efficiency at FOAM is only 38%, due to diffusion and bioirrigation across a high gradient of dissolved phosphate near the sediment-seawater interface. This is comparable to observations from California continental slope sediments, where 75% of the phosphate generated by the remineralization of iron hydroxides and organic matter is released to seawater[16].

The ability of our model to reproduce authigenic P and relevant pore-water profiles measured at FOAM suggests that the reaction rate law for CFA formation used in this study is mechanistically accurate, in spite of continuing uncertainties regarding the precise chemical formulation of CFA and K_{spCFA}. Previous modeling exercises have described the kinetics of CFA formation with an equilibrium phosphate concentration term[17-20]. However, experimental studies have shown that rates of crystal growth of calcium hydroxylapatite and calcium fluorapatite are functions of saturation state[21,22]. The reaction rate constant for the baseline model run (Table S5) is 2.7×10^{-8} M yr^{-1}, consistent with sluggish precipitation kinetics.

Application to ODP Site 846/1226

We have also applied the same model to a deep water setting (ODP site 846/1226), where P species and other geochemical variables have been measured[23-26]. In this site, the early diagenetic sink-switch from organic P and iron-bound P to CFA occurs from the top of the sediment pile to about 20 m below the sediment-seawater interface (Fig. S3), equivalent to a timescale of about 0.5 Myr[25]. For this exercise, model parameters were adjusted to be

consistent with slow sedimentation rates and low organic matter fluxes. The results are shown in Fig. S3. Our model can reproduce sediment profiles for ODP site 846/1226, including P species (Fig. S3). The ability of our model to reproduce both shallow marine and deep-sea sediment data provides strong support that our parameterizations and mechanistic framework is robust.

2. Deep-Sea Sedimentary P Speciation Compilation

To better ground our model results, we have compiled P speciation data for deep-sea sediments spanning the last 80 myr of the Phanerozoic[25-30]. The compiled data include 17 ODP sites from the Pacific, Atlantic and Indian Oceans, and thus do not have strong regional biases. We have removed the data for the last 2–6 myr as it has been determined that the diagenetic "sink-switch" from organic P and iron-bound P to CFA may still, during this interval of early diagenesis, be ongoing[19,25]. It has been previously documented that pyrite oxidation during sample storage can lead to underestimation of the CFA content of carbonate-poor sediments[31]. However, it is unlikely that this process strongly influenced our compiled data because 1) the deep-sea sediments from which we compiled these data are not pyrite-rich, due to slow sulfate reduction rates or even the absence of sulfate reduction (as indicated by porewater sulfate concentration profiles); and 2) most of the deep-sea sediments from which these data were compiled are carbonate-rich (>20%), which precludes the dissolution of CFA by pyrite oxidation[31]. Our compiled data show a gradual decrease in the burial ratio of CFA-associated P and total reactive P (a sum of organic P, CFA and iron-bounded P) ($P_{CFA}/P_{Reactive}$) from ~35 Ma onward (Fig. 4), which is in phase with a decrease in marine Ca concentrations (and an increase in the Mg/Ca value of seawater, ref. 32-34).

3. Coupled Carbon-Phosphorus-Oxygen Cycle Model

As an alternative means of gauging if changes in the Ca cycle will shape the global P and C cycles, we forced our global carbon cycle model with previously estimated, independently derived values for seawater DIC, seawater dissolved Ca and Mg concentrations, seawater pH, and bioturbation intensities through the Phanerozoic[32,35-37]. Bottom-water oxygen concentrations and organic matter loading vary with time-dependent

pO_2 and marine P concentrations. Following previous work, we also included a plant-assisted weathering effect (following ref. 38). Given model simplifications, our goal was not to provide the 'best' estimate of Phanerozoic pO_2—our aim was, more simply, to explore whether Ca-driven shifts in P cycling—as predicted by empirical records and our model-based investigations—would drive significant changes in surface O_2 levels.

We compiled the results of time-dependent runs of our diagenetic model in order to build look-up tables with a time step of 1 myr from 540 Ma to 0 Ma. For each run, environmental parameters such as [DIC], [Ca], [Mg] and pH of marine bottom waters and the bioturbation parameters were varied temporally to match empirical estimates for these values derived from Phanerozoic geologic archives, using data adopted from the literature (see Supplementary Table 7). Although there are estimates for bottom-water [DIC] variation through the Cenozoic vary (e.g., ref. 39), a change in the input value of bottom-water [DIC] will not substantially influence the model results, as bottom-water [DIC] does not strongly influence CFA burial (see Fig. 3). We carried out a series of model runs with different bottom-water oxygen concentrations and organic carbon fluxes to the sediment-seawater interface to build look-up tables for the deep sea and shallow oceans, respectively (with the parameters shown in Supplementary Table 8), which were then used to force P burial at each time step.

The default model output is shown in Fig. S11 and S12. Variation in calcium-bound P (CFA) burial largely follows the long-term evolution of seawater dissolved Ca concentrations, which are characterized by a strong inverse correlation with organic carbon burial and atmospheric oxygen levels. Increases in the calcium-bound P burial flux are correlated with decreases in the size of the total marine P reservoir (M5), which, in turn, will decrease organic carbon burial—facilitating a drop in O_2 levels. Decreases in the calcium-bound P (CFA) burial flux will, conversely, lead to increases in the size of the total marine P reservoir (M5), which will increase organic carbon burial, leading to increases in O_2 level. Therefore, temporal shifts in the magnitude of the calcium-bound P burial flux will, as determined by the long-term evolution of seawater Ca concentration, play a significant role in controlling atmospheric O_2 levels through the Phanerozoic.

To further determine which factors most strongly influence model outputs, we carried out sensitivity tests (Fig. S12). Our default model interpolated outputs from our diagenetic model, using a time step of 1 million years. The flux of terrestrial non-reactive phosphorus to marine reactive phosphorus (F25) could also be influenced by the magnitude of plant-assisted weathering (see ref. 38). Based on the temporal evolution of land plants (Table S13), we include a plant-assisted weathering effect in our default run, following Eq. S15:

$$F_{25} = k_{2-5} \cdot M_2 \cdot (1 + ap * \ Plant_{evolution})/(1 + ap) \qquad \text{(S15)}$$

where ap is a non-dimensional factor, which was set as 0.5 in this study. We have also performed runs without the effect of plant-assisted weathering, for comparison. As shown in Fig. S12, plant-assisted weathering could have increased the magnitude of fluctuations in atmospheric O_2 levels from the mid-Paleozoic onward (though incorporation of plant-assisted weathering does not change the shape of the predicted Phanerozoic O_2 curve). Given uncertainties in seawater dissolved Ca concentration estimates, we have also performed a set of runs with offsets (±2.5 mM) from the baseline Ca concentration (Fig. S12a), which are shown as the upper and lower boundaries of the bars in Fig. S12b and S12c. These results show that $p\mathrm{O}_2$ is higher at low seawater dissolved Ca concentrations (Fig. S12).

The results of this coupled modeling exercise (Fig. S12) indicate that Phanerozoic CFA burial fluxes largely follow contemporaneous variations in seawater dissolved Ca concentrations. This indicates that, over the course of both the Paleozoic (coeval with the rise of bioturbation) and the Phanerozoic as a whole, marine Ca concentrations played a key role in regulating the efficiency of CFA burial (and thus P burial as a whole). Our coupled global model also suggests that major swings in CFA and P burial result in major swings in atmospheric oxygen levels. Considering a global oxygen cycle with only the redox and nutrient feedbacks in the utilized model, Variations in marine Ca concentrations could have caused shifts in atmospheric *p*O2 from less than 10% to greater than 25% (v/v), encompassing essentially the full range of values previously predicted for the Phanerozoic as a whole[38,40,41].

Our results offer new insights into the factors driving major shifts in Phanerozoic atmospheric pO_2. Fluid-inclusion data suggest an increase in seawater dissolved Ca concentrations during the early Cambrian[32], coincident with a previously suggested interval of ocean deoxygenation (e.g., refs. 42, 43). These data also indicate low seawater dissolved Ca concentrations during the Carboniferous–Permian[32,44], which would have inhibited CFA formation and driven the development of the high atmospheric pO_2 that, on the basis of both geochemical and paleontological archives[40,41,45], has been previously suggested to be characteristic of this time (interval 3 of Fig. S12c). Atmospheric pO_2 values during the Mesozoic and the Cenozoic are currently poorly resolved[46], making it difficult to gauge whether shifts in marine dissolved Ca concentrations and atmospheric oxygen levels were coupled over this interval (Fig. S13). Nonetheless, our framework suggests that large shifts in marine dissolved Ca concentrations, as indicated by the fluid inclusion record, would have driven significant changes in marine P and atmospheric oxygen levels—consistent with most pO_2 reconstructions.

Although debated, increases in organic carbon burial tied to diversification of the terrestrial biosphere are commonly considered to have played a substantial role in driving Earth's protracted oxygenation[38,40,41,45,47]. Our results do not preclude the possible importance of the rise of land plants in shaping atmospheric pO_2. However, they suggest that seawater dissolved Ca concentration may have been an equally important factor influencing surface oxygen levels over the duration of the Phanerozoic. In addition, our proposed link between marine Ca and atmospheric O_2 should have been operative throughout Earth's history. Although terrestrial organic carbon burial has recently received much attention[38,40,41] and has been treated as a key factor driving pO_2 increases from the Ordovician onward, the "real" magnitude of this flux through the Phanerozoic has been questioned (e.g., ref. 47). Also, it has been suggested that the ratio between land plant-derived kerogen and total kerogen is only weakly correlated with the sedimentary organic carbon to pyrite sulfur ratio[48]. In fact, it is difficult to robustly reconstruct the magnitude of this flux, due to the relative paucity of appropriate archives of terrestrial organic carbon (e.g., coal). Interestingly, as shown in Fig. S13, our model can generate elevated Carboniferous–Permian O_2 levels comparable to those predicted by other (carbon isotope based) models, without incorporation of a major terrestrial organic carbon flux. Further, our model results

are broadly consistent with recent COPSE predictions for the Paleozoic O_2 despite different forcings—indicting the potential for compounding effects in drivers of pO_2 evolution (Fig. S13).

There are, of course, a wide range of factors which may influence O_2 levels (e.g., paleoceanographic circulation, sulfur cycling, fire feedback and uplift), not all of which are considered in detail here. The goal of our model is not to replace current holistic carbon and oxygen cycle models (e.g., COPSE, CANOPS, MAGic). However, our model provides valuable insight into the interaction between diagenetic and ocean-scale processes regulating key nutrient cycles such as the P cycle. Our modeling exercise demonstrates that, although historically underappreciated, seawater Ca concentrations are a key factor shaping the Phanerozoic carbon-oxygen and phosphorus cycles.

Given widely accepted tectonic controls on marine cation concentrations (e.g., Ref. 49), our results suggest a strong coupling between the solid Earth, the marine P cycle, and atmospheric pO_2. Although a number of factors control marine dissolved Ca concentrations, it is generally accepted that the extent of Mg/Ca exchange in hydrothermal systems plays a major role in controlling seawater cation ratios (e.g., Refs. 49, 50). It has been suggested that high seafloor spreading and volcanogenic outgassing rates would elevate atmospheric pCO_2 and increase the weathering flux of nutrients to the ocean. Our model predicts that increased spreading rates would not only elevate atmospheric pCO_2, but also promote greater CFA burial by mediating increased seawater dissolved Ca concentrations, ultimately driving a decrease in atmospheric pO_2 (Fig. S14).

Supplementary Tables

Supplementary Table 1. Chemical species included in the model and their boundary conditions for the FOAM and ODP 1226 site.

Solids (mmol cm^{-2} yr^{-1})		
Variables	FOAM Values	ODP 1226 values
Organic carbon (orgC)	0.3	0.037
Organic nitrogen (orgN)	Linked with orgC	Linked with orgC
Organic phosphorus (orgP)	Linked with orgC	Linked with orgC
Adsorbed Fe (Surf-Fe^{+})	0	0
Highly reactive Fe hydroxides ($Fe(OH)_3^{\alpha}$)	0.00685	0.000685
Less reactive Fe hydroxides ($Fe(OH)_3^{\beta}$)	0	0
Unreactive Fe hydroxides ($Fe(OH)_3^{\gamma}$)	0	0
Magnetite (Fe_3O_4)	0.00199	0
Biotite (Biot)	0.004	0.0004
Highly reactive Mn oxide (MnO_2^{α})	0.00327	0.000327
Less reactive Mn oxide (MnO_2^{β})	0	0
Phosphate associated with $Fe(OH)_3^{\alpha}$ (P_{Fe}^{α})	Linked with $Fe(OH)_3^{\alpha}$	Linked with $Fe(OH)_3^{\alpha}$
Phosphate associated with $Fe(OH)_3{}^{\beta}$ (P_{Fe}^{β})	0	0
Phosphate associated with $Fe(OH)_3{}^{\gamma}$ (P_{Fe}^{γ})	0	0
Pyrite (FeS_2)	0	0
Iron monosulfide (FeS)	0	0
Elemental sulfur (S_0)	0	0
Aragonite (Arag)	0	0
Calcite (Calc)	0.432	0.0432
Rhodochrosite (Rhod)	0	0
Carbonate fluorapatite (CFA)	5.27×10^{-5}	5.27×10^{-6}
Vivianite (Vivi)	0	0
**Solutes (mmol cm^{-3})**		
Oxygen (O_2)	0.15×10^{-3}	0.15×10^{-3}
Nitrate (NO_3^-)	11.8×10^{-6}	31.6×10^{-6}
Sulfate (SO_4^{2-})	22×10^{-3}	28×10^{-3}
Ammonium and ammonia (ΣNH_4^+)	0	0

Inorganic carbon (ΣCO_3^{2-})	2×10^{-3}	2.25×10^{-3}
Hydrogen sulfide (ΣH_2S)	0	0
Phosphate (ΣPO_4^{3-})	1×10^{-6}	2.4×10^{-6}
Methane (CH_4)	0	0
Ferrous iron (Fe^{2+})	0	0
Manganese (Mn^{2+})	0	0
Calcium (Ca^{2+})	8.6×10^{-3}	10×10^{-3}
Hydrogen (H^+)	$10^{-7.64}$	$10^{-7.87}$
Fluoride (F^-)	70×10^{-6}	70×10^{-6}
Sodium (Na^+)	380.56×10^{-3}	481×10^{-3}
Magnesium (Mg^{2+})	46×10^{-3}	53×10^{-3}

Note:

1). the fluxes for all the component are fixed as zero at the lower boundary.

2). The flux ratios of $P_{Fe}/Fe(OH)_3$ are fixed as γ (Table S4) for all three components

3). The fluxes of orgN and orgP are controlled by the flux of orgC and r_N and r_P (Table S4)

4). The boundary conditions of the solutes are from refs. 1-9.

Supplementary Table 2. Reactions considered in the diagenetic model.

Number	Equations
**Primary redox reactions:**	
R1	$(CH_2O)(NH_3)_x(H_3PO_4)_y + (1+2x)O_2 \rightarrow CO_3^{2-} + xNO_3^- + (2+x+3y)H^+ + yPO_4^{3-} + (x+1)H_2O$
R2	$(CH_2O)(NH_3)_x(H_3PO_4)_y + \left(\frac{4+3x}{5}\right)NO_3^- + H_2O \rightarrow CO_3^{2-} + \left(\frac{6+15y-3x}{5}\right)H^+ + \left(\frac{2+4x}{5}\right)N_2 + yPO_4^{3-} + \frac{7+9x}{5}H_2O$
R3	$(CH_2O)(NH_3)_x(H_3PO_4)_y + 2MnO_2^{\alpha} + (2-3y)H^+ \rightarrow CO_3^{2-} + 2Mn^{2+} + xNH_3 + yPO_4^{3-} + 2H_2O$
R4[a]	$(CH_2O)(NH_3)_x(H_3PO_4)_y + 4Fe(OH)_3^{\alpha} + 4\theta P_{Fe}^{\alpha} + (6-3y-12r)H^+ \rightarrow CO_3^{2-} + 4Fe^{2+} + xNH_3 + (y + 4\theta)PO_4^{3-} + 10H_2O$
R5	$(CH_2O)(NH_3)_x(H_3PO_4)_y + \frac{1}{2}SO_4^{2-} \rightarrow CO_3^{2-} + (\frac{3}{2} + 3y)H^+ + \frac{1}{2}HS^- + xNH_3 + yPO_4^{3-}$
R6	$(CH_2O)(NH_3)_x(H_3PO_4)_y + \frac{1}{2}H_2O \rightarrow \frac{1}{2}CO_3^{2-} + (1+3y)H^+ + \frac{1}{2}CH_4 + xNH_3 + yPO_4^{3-}$
**Secondary reactions:**	
R7	$NH_3 + 2O_2 \rightarrow NO_3^- + H^+ + H_2O$
R8	$Mn^{2+} + 0.5O_2 + H_2O \rightarrow MnO_2^{\alpha} + 2H^+$
R9	$Fe^{2+} + 0.25O_2 + 2.5H_2O + \gamma PO_4^{3-} \rightarrow Fe(OH)_3^{\alpha} + \gamma P_{Fe}^{\alpha} + (2-3r)H^+$
R10	$FeS + 2O_2 \rightarrow SO_4^{2-} + Fe^{2+}$
R11	$FeS_2 + 3.5O_2 + H_2O \rightarrow 2SO_4^{2-} + Fe^{2+} + 2H^+$
R12	$HS^- + 2O_2 \rightarrow SO_4^{2-} + H^+$
R13	$CH_4 + 2O_2 \rightarrow CO_3^{2-} + H_2O + 2H^+$
R14	$MnO_2^{\alpha,\beta} + 2Fe^{2+} + 2\gamma PO_4^{3-} + 4H_2O \rightarrow 2Fe(OH)_3^{\alpha} + 2\gamma P_{Fe}^{\alpha} + Mn^{2+} + (2-6r)H^+$
R15	$MnO_2^{\alpha,\beta} + HS^- + 3H^+ \rightarrow Mn^{2+} + S_0 + 2H_2O$
R16	$2Fe(OH)_3^{\alpha,\beta} + 2\theta P_{Fe}^{\alpha,\beta} + HS^- + (5-6r)H^+ \rightarrow 2Fe^{2+} + 2\theta PO_4^{3-} + S_0 + 6H_2O$
R17	$Fe^{2+} + HS^- \rightarrow FeS + H^+$
R18	$SO_4^{2-} + CH_4 \rightarrow HS^- + H_2O + CO_3^{2-} + H^+$
R19	$4S_0 + 4H_2O \rightarrow 3HS^- + SO_4^{2-} + 5H^+$
R20	$FeS + S_0 \rightarrow FeS_2$
R21	$Fe(OH)_3^{\alpha} + \theta P_{Fe}^{\alpha} \rightarrow Fe(OH)_3^{\beta} + \theta P_{Fe}^{\beta}$
R22	$MnO_2^{\alpha} \rightarrow MnO_2^{\beta}$
R23	$Ca^{2+} + CO_3^{2-} \rightarrow$ Aragonite
R24	$Ca^{2+} + CO_3^{2-} \rightarrow$ Calcite
R25	$Mn^{2+} + CO_3^{2-} \rightarrow MnCO_3$
R26	$9.54Ca^{2+} + 0.33Na^+ + 0.13Mg^{2+} + 4.8PO_4^{3-} + 1.2CO_3^{2-} + 2.48F^- \rightarrow Ca_{9.54}Na_{0.33}Mg_{0.13}(PO_4)_{4.8}(CO_3)_{1.2}F_{2.48}$
R27	$FeS + HS^- + H^+ \rightarrow FeS_2 + H_2$
R28	$KMgFe_2AlSi_3O_{10}(OH)_2 + 7H^+ + 0.5H_2O \rightarrow K^+ + Mg^{2+} + 2Fe^{2+} + 0.5Al_2Si_2O_5(OH)_4 + 2H_4SiO_4$
R29	$Fe_3O_4 + HS^- + 7H^+ \rightarrow 3Fe^{2+} + S_0 + 4H_2O$
R30	$Surf-H + Fe^{2+} \rightarrow Surf-Fe^+ + H^+$
R31	$Surf-Fe^+ + 0.25O_2 + \gamma PO_4^{3-} + 2.5H_2O \rightarrow Surf-H + Fe(OH)_3^{\alpha} + \gamma P_{Fe}^{\alpha} + (1-3r)H^+$
R32	$3Fe^{2+} + 2H_2PO_4^- \rightarrow Fe_3(PO_4)_2 + 4H^+$

Reversible acid-base reactions

$CO_2(aq) + H_2O \rightarrow H^+ + HCO_3^-$

$HCO_3^- \rightarrow H^+ + CO_3^{2-}$

$NH_4^+ \rightarrow H^+ + NH_3$

$H_2PO_4^- \rightarrow H^+ + HPO_4^{2-}$

$HPO_4^{2-} \rightarrow H^+ + PO_4^{3-}$

$H_2S \rightarrow H^+ + HS^-$

Note:

a. $\theta = P_{Fe}^{\alpha}/Fe(OH)_3^{\alpha}$ and/or $P_{Fe}^{\beta}/Fe(OH)_3^{\beta}$

Supplementary Table 3. Reaction rate laws for the diagenetic model.

Symbol	Expressions
R1	$k_i orgC_i(\frac{[O_2]}{[O_2]_{lim}+[O_2]})$
R2	$k_i orgC_i(\frac{[O_2]_{lim}}{[O_2]_{lim}+[O_2]})(\frac{[NO_3^-]}{[NO_3^-]_{lim}+[NO_3^-]})$
R3	$k_i orgC_i(\frac{[O_2]_{lim}}{[O_2]_{lim}+[O_2]})(\frac{[NO_3^-]_{lim}}{[NO_3^-]_{lim}+[NO_3^-]})(\frac{[MnO_2^{\alpha}]}{[MnO_2]_{lim}+[MnO_2^{\alpha}]})$
R4	$k_i orgC_i(\frac{[O_2]_{lim}}{[O_2]_{lim}+[O_2]})(\frac{[NO_3^-]_{lim}}{[NO_3^-]_{lim}+[NO_3^-]})(\frac{[MnO_2]_{lim}}{[MnO_2]_{lim}+[MnO_2^{\alpha}]})(\frac{[Fe(OH)_3^{\alpha}]}{[Fe(OH)_3]_{lim}+[Fe(OH)_3^{\alpha}]})$
R4P	$k_i orgC_i(\frac{[O_2]_{lim}}{[O_2]_{lim}+[O_2]})(\frac{[NO_3^-]_{lim}}{[NO_3^-]_{lim}+[NO_3^-]})(\frac{[MnO_2]_{lim}}{[MnO_2]_{lim}+[MnO_2^{\alpha}]})(\frac{[P_{Fe}^{\alpha}]}{[Fe(OH)_3]_{lim}+[Fe(OH)_3^{\alpha}]})$
R5	$a_{SO_4^{2-}}\text{-}k_i orgC_i(\frac{[O_2]_{lim}}{[O_2]_{lim}+[O_2]})(\frac{[NO_3^-]_{lim}}{[NO_3^-]_{lim}+[NO_3^-]})(\frac{[MnO_2]_{lim}}{[MnO_2]_{lim}+[MnO_2^{\alpha}]})(\frac{[Fe(OH)_3]_{lim}}{[Fe(OH)_3]_{lim}+[Fe(OH)_3^{\alpha}]})(\frac{[SO_4^{2-}]}{[SO_4^{2-}]_{lim}+[SC}$ [illegible]
R6	$a_{SO_4^{2-}}\text{-}k_i orgC_i(\frac{[O_2]_{lim}}{[O_2]_{lim}+[O_2]})(\frac{[NO_3^-]_{lim}}{[NO_3^-]_{lim}+[NO_3^-]})(\frac{[MnO_2]_{lim}}{[MnO_2]_{lim}+[MnO_2^{\alpha}]})(\frac{[Fe(OH)_3]_{lim}}{[Fe(OH)_3]_{lim}+[Fe(OH)_3^{\alpha}]})(\frac{[SO_4^{2-}]_{lim}}{[SO_4^{2-}]_{lim}+[SC}$ [illegible]
ROC [a]	R1 + R2 + R3 + R4 + R5 + R6
R7	$k7 \cdot O_2 \cdot \Sigma NH_4^+$
R8	$k8 \cdot O_2 \cdot Mn^{2+}$
R9	$k9 \cdot O_2 \cdot Fe^{2+}$
R9P	$k9 \cdot O_2 \cdot Fe^{2+} \cdot \gamma \cdot \sum PO_4^{3-} / (\sum PO_4^{3-} + [PO_4^{3-}]_{lim})$
R10	$k10 \cdot O_2 \cdot FeS$
R11	$k11 \cdot O_2 \cdot FeS_2$
R12	$k12 \cdot O_2 \cdot \Sigma H_2S$
R13	$k13 \cdot O_2 \cdot CH_4$
R14α	$k14 \cdot MnO_2^{\alpha} \cdot Fe^{2+}$
R14αP	$k14 \cdot MnO_2^{\alpha} \cdot Fe^{2+} \cdot \gamma \cdot \sum PO_4^{3-} / (\sum PO_4^{3-} + [PO_4^{3-}]_{lim})$
R14β	$k14 \cdot MnO_2^{\beta} \cdot Fe^{2+}$
R14βP	$k14 \cdot MnO_2^{\beta} \cdot Fe^{2+} \cdot \gamma \cdot \sum PO_4^{3-} / (\sum PO_4^{3-} + [PO_4^{3-}]_{lim})$
R15α	$k15 \cdot MnO_2^{\alpha} \cdot \Sigma H_2S$
R15β	$k15 \cdot MnO_2^{\beta} \cdot \Sigma H_2S$
R16α	$k16 \cdot Fe(OH)_3^{\alpha} \cdot \Sigma H_2S$
R16αP	$k16 \cdot P_{Fe}^{\alpha} \cdot \Sigma H_2S$
R16β	$k16 \cdot Fe(OH)_3^{\beta} \cdot \Sigma H_2S$
R16βP	$k16 \cdot P_{Fe}^{\beta} \cdot \Sigma H_2S$
R17	$k17 \cdot Fe^{2+} \cdot \Sigma H_2S$
R18	$k18 \cdot \Sigma SO_4 \cdot CH_4$
R19	$k19 \cdot S_0$
R20	$k20 \cdot FeS \cdot S_0$
R21	$k21 \cdot Fe(OH)_3^{\alpha}$

R21P	$k21 \cdot P_{Fe}^{\alpha}$
R22	$k22 \cdot MnO_2^{\alpha}$
R23	$k23^{+} \cdot (\Omega_{arga}-1)$ $\Omega_{arga}>1$ $k23^{-} \cdot (\Omega_{arga}-1) \cdot arag$ $\Omega_{arga}<1$
R24	$k24^{+} \cdot (\Omega_{calc}-1)$ $\Omega_{calc}>1$ $k24^{-} \cdot (\Omega_{calc}-1) \cdot calc$ $\Omega_{calc}<1$
R25	$k25^{+} \cdot (\Omega_{rhod}-1)$ $\Omega_{rhod}>1$ $k25^{-} \cdot (\Omega_{rhod}-1) \cdot rhod$ $\Omega_{rhod}<1$
R26	$k26 \cdot (\Omega_{CFA}-1)$ $\Omega_{CFA}>1$ 0 $\Omega_{CFA}<1$
Ω_{arag}	$([CO_3^{2-}] \cdot [Ca^{2+}])/Ksp_{arag}$
Ω_{calc}	$([CO_3^{2-}] \cdot [Ca^{2+}])/Ksp_{calc}$
Ω_{rhod}	$([CO_3^{2-}] \cdot [Mn^{2+}])/Ksp_{rhod}$
Ω_{CFA}	$\frac{([Ca^{2+}] \cdot rCa)^{9.54} \cdot ([Na^{+}] \cdot rNa)^{0.33} \cdot ([Mg^{2+}] \cdot rMg)^{0.13} \cdot ([PO_4^{3-}] \cdot rPO_4)^{4.8} \cdot ([CO_3^{2-}] \cdot rCO_3)^{1.2} \cdot ([F^{-}] \cdot rF)^{2.48}}{K_{spCFA}}$
Ksp_{CFA}	$10^{-83.231+2.3307 \cdot \log([CO_3^{2-}] \cdot rCO_3)}$
R27	$k27 \cdot FeS \cdot \Sigma H_2S$
R28 [b]	$k28 \cdot biot_0$
R29	$K29 \cdot Fe_3O_4 \cdot (\Sigma H_2S)^{0.5}$
R30	$k30 \cdot Surf\text{-}Fe^{+} \cdot O_2$
R30P	$k30 \cdot Surf\text{-}Fe^{+} \cdot O_2 \cdot \gamma \cdot \sum PO_4^{3-} / (\sum PO_4^{3-} + [PO_4^{3-}]_{lim})$
R31	$k31 \cdot [Fe^{2+}]/([Fe^{2+}]+[Fe^{2+}]_{lim}) \cdot [HPO_4^{2-}]/([HPO_4^{2-}] + [HPO_4^{2-}]_{lim})$

Note:

a. the decomposition rate of organic matter

b. $biot_0$ is the concentration of biotite at the sediment-seawater interface

Supplementary Table 4: Reaction rate for each component

Variables	Reaction rates
orgC	-ROC
orgN	-ROC*r_N
orgP	-ROCa*r_{P1}-ROCb*r_{P2}
$Fe(OH)_3^{\alpha}$	- 4*R4 + 1/f*R9 + 2*(R14α+ R14β) - 2*R16α – R21 + R30
$Fe(OH)_3^{\beta}$	- 2*R16β + R21
$Fe(OH)_3^{\gamma}$	0
Fe_3O_4	-R29
Biot	-R28
MnO_2^{α}	- 2*R3 + 1/f*R8 – R14α – R15α - R22
MnO_2^{β}	- R14β - R15β + R22
P_{Fe}^{α}	- 4*R4P +1/f*R9P + 2*(R14αP+ R14βP) - 2*R16αP – R21P + R30P
P_{Fe}^{β}	- 2*R16βP + R21P
P_{Fe}^{γ}	0
FeS_2	- R11 + R20 + R27
FeS	- R10 + 1/f*R17 - R20 - R27
S_0	+ (R15α+R15β) + (R16 α+R16β) - 4*R19 - R20 + R29
Arag	+ R23
Calc	+ R24
Rhod	+ R25
CFA	+ R26
Vivi	+ R31
O_2	- (1+2*r_N)*f*R1 -2*R7 - 0.5*R8 - 0.25*R9 - 2*f*R10 - 3.5*f*R11 - 2*R12 - 2*R13 - 0.25*f*R30
NO_3^-	+ r_N*f*R1 - (0.8+0.6*r_N)*f*R2 + R7
SO_4^{2-}	- 0.5*f*R5+ f*R10 + 2*f*R11 + R12 - R18 + f*R19
ΣNH_4^+	+ f* r_N *(R3+R4+R5+R6) - R7
ΣCO_3^{2-}	+ f *(ROC - 0.5*R6) + R13 + R18 - f*R23 - f *R24 - f *R25 - 1.2*f *R26
ΣH_2S	+ 0.5*f *R5 - R12 - f *(R15α+R15β) - f *(R16 α+R16β) - R17 + R18 + 3*f *R19 - f *R27 - f *R29
ΣPO_4^{3-}	+ f *(ROCa*r_{P1}+ROCb*r_{P2}) - 4.8*f *R27 + 4*f *R4P – R9P - 2* f.grid*(R14αP+ R14βP) + 2*f *(R16αP+R16βP) - f.grid*R30P
CH_4	+ 0.5*f *R6 - R13 - R18
Fe^{2+}	+1/(1+kFe)*(4*f *R4 - R9 + f *R10 + f *R11 - 2*f *(R14α+ R14β) + 2*f *(R16α+R16β) - R17 + 2*f *R28 + 3*f *R29) + 1/(1+kFe)*f*(-R30)
Mn^{2+}	+ 2*f *R3 - R8 + f *(R14α+ R14β) + f *(R15α+R15β) - f *R25
Ca^{2+}	- f *R23 - f *R24 - 9.54*f *R26
TP	+ (2+r_N)*f *R1 + 1.2*f *R2 - 0.6*f *R2*r_N - 2*f *R3 - 6*f *R4 + 1.5*f *R5 + f*R6 + 3*f *(ROCa*r_{P1}+ROCb*r_{P2}) + 12*f*R4P + R7 + 2*R8 + (2-3*γ*SumH2PO4/(SumH2PO4+KPO4))*R9 + 2*f *R11 + R12 + 2*R13 + (2-6* γ *SumH2PO4/(SumH2PO4+KPO4))*f *(R14α+ R14β) - 3*f*(R15α+R15β) -

	5*f*(R16α+R16β) + 6*f *(R16αP+ R16βP) + R17 + R18 + 5*f*R19 - f*R26 - 7*f*R27 - 7*f*reac28 + (1-3* γ *SumH2PO4/(SumH2PO4+KPO4))*f *R29 + kFe/(1+kFe)*RTFeII - 1/(1+kFe)*f*RTSurfFe
F^-	- 2.48*f *R26
Na^+	- 0.33*f *R26
Mg^{2+}	- 0.13*f *R26

Supplementary Table 5. Model parameters for the FOAM site.

symbol	description	Value	unit	Sources
L	The length of domain	300	cm	This study
T	Marine bottom-water temperature	12	^{o}C	ref. 3
S	Marine bottom-water salinity	28.4	1	ref. 4
P	Marine bottom-water pressure	2	bar	ref. 8
ϕ_0	Porosity at surface	0.64	1	refs. 2,51
ϕ_∞	Porosity at depth	0.64	1	refs. 2,51
λ	Porosity attenuation length	5	cm	This study
ρ	Sediment density	2.5	g cm^{-3}	ref. 2
ω_0	Sediment accumulation rate	0.2	cm yr^{-1}	ref. 6
D_{B0}	Biodiffusion coefficient at surface	10^{-7}	cm^2 s^{-1}	This study
xbt	Biodiffusion attenuation coefficient	3	cm	This study
a_0	Bioirrigation coefficient at surface	100	yr^{-1}	This study
xbi	Bioirrigation attenuation coefficient	0.8	cm	This study
γ	P/Fe ratio for iron-bound P	0.24	1	This study
r_Fe	Bioirrigation scaling coefficient of Fe^{2+}	0	1	ref. 52
r_Mn	Bioirrigation scaling coefficient of Mn^{2+}	0.2	1	ref. 52
a	Average lifetime of more reactive orgC	0.15	yr	This study
v	Shape of orgC distribution	0.12	1	This study
r_N	C/N molar ratio in organic matter	106:10	mol mol^{-1}	This study
r_{P1}	C/P molar ratio in organic matter α	106:1.3	mol mol^{-1}	This study
r_{P2}	C/P molar ratio in organic matter β	106:0.27	mol mol^{-1}	This study
ε	accelerated factor for P regeneration rate in organic matter α	1	1	This study
$[O_2]_{lim}$	Limiting concentration of O2	20	μM	refs. 53,54
$[NO_3^-]_{lim}$	Limiting concentration of NO_3^-	4	μM	refs. 53,54
$[MnO_2]_{lim}$	Limiting concentration of MnO_2	32	μmol g^{-1}	refs. 53,54
$[Fe(OH)_3]_{lim}$	Limiting concentration of $Fe(OH)_3$	65	μmol g^{-1}	refs. 53,54
$[SO_4^{2-}]_{lim}$	Limiting concentration of SO_4^{2-}	1.6	mM	refs. 53,54
$[PO_4^{3-}]_{lim}$	Limiting concentration of PO_4^{3-} uptake into $Fe(OH)_3^{\alpha}$	10	μM	ref. 17
$a_{SO_4^{2-}}$	Attenuation factor for SO_4^{2-} reduction	0.2	1	ref. 54
k7	Rate constant of R7	1×10^7	M^{-1} yr^{-1}	ref. 53

k8	Rate constant of R8	6.94×10^{5}	$M^{-1}\ yr^{-1}$	ref. 52
k9	Rate constant of R9	1.4×10^{8}	$M^{-1}\ yr^{-1}$	ref. 53
k10	Rate constant of R10	3×10^{5}	$M^{-1}\ yr^{-1}$	ref. 53
k11	Rate constant of R11	1.89×10^{4}	$M^{-1}\ yr^{-1}$	ref. 52
k12	Rate constant of R12	1.6×10^{5}	$M^{-1}\ yr^{-1}$	ref. 53
k13	Rate constant of R13	10^{10}	$M^{-1}\ yr^{-1}$	ref. 53
k14	Rate constant of R14	3×10^{6}	$M^{-1}\ yr^{-1}$	ref. 53
k15	Rate constant of R15	2×10^{4}	$M^{-1}\ yr^{-1}$	ref. 53
k16	Rate constant of R16	8×10^{3}	$M^{-1}\ yr^{-1}$	ref. 53
k17	Rate constant of R17	1.48×10^{6}	$M^{-1}\ yr^{-1}$	ref. 52,54
k18	Rate constant of R18	10^{4}	$M^{-1}\ yr^{-1}$	ref. 53
k19	Rate constant of R19	3.16	yr^{-1}	ref. 52
k20	Rate constant of R20	7.26×10^{3}	$M^{-1}\ yr^{-1}$	ref. 52
k21	Rate constant of R21	0.57	yr^{-1}	ref. 52
k22	Rate constant of R22	1.7	yr^{-1}	ref. 52
$k23^{+}$	Rate constant for the formation of arag	3×10^{-6}	$M\ yr^{-1}$	This study
$k23^{-}$	Rate constant for the dissolution of arag	0.5	yr^{-1}	ref. 55
$k24^{+}$	Rate constant for the formation of calc	3×10^{-7}	$M\ yr^{-1}$	This study
$k24^{-}$	Rate constant for the dissolution of calc	0.5	yr^{-1}	ref. 55
$k25^{+}$	Rate constant for the formation of rhod	3×10^{-6}	$M\ yr^{-1}$	ref. 53
$k25^{-}$	Rate constant for the dissolution of rhod	0.25	yr^{-1}	ref. 53
k26	Rate constant of R26	2.7×10^{-8}	$M\ yr^{-1}$	This study
k27	Rate constant of R27	3.25×10^{3}	$M^{-1}\ yr^{-1}$	ref. 56
k28	Rate constant of R28	3×10^{-4}	yr^{-1}	This study
k29	Rate constant of R29	5.4×10^{-3}	$mM^{-0.5}\ yr^{-1}$	ref. 1
k30	Rate constant of R30	5×10^{6}	$M^{-1}\ yr^{-1}$	ref. 53
k31	Rate constant of R31	1×10^{-6}	$M^{-1}\ yr^{-1}$	ref. 57
$[Fe^{2+}]_{lim}$	Half-saturation constant for $[Fe^{2+}]$	5×10^{-6}	M	ref. 58
$[HPO_4^{2-}]_{lim}$	Half-saturation constant for $[HPO_4^{2-}]$	5×10^{-6}	M	ref. 58
k_{Fe}	Adsorption coefficient for Fe^{2+}	500	1	ref. 53
Ksp_{arag}	Apparent equilibrium constant of arag	5.6×10^{-7}	M^2	ref. 59
Ksp_{calc}	Apparent equilibrium constant of calc	3.52×10^{-7}	M^2	ref. 59
Ksp_{rhod}	Apparent equilibrium constant of rhod	10^{-9}	M^2	ref. 60
rCa	Activity coefficient for Ca^{2+}	0.219	1	a
rNa	Activity coefficient for Na^{+}	0.657	1	a
rMg	Activity coefficient for Mg^{2+}	0.282	1	a

rPO_4	Activity coefficient for PO_4^{3-}	3.7×10^{-5}	1	ref. 61
rCO_3	Activity coefficient for CO_3^{2-}	0.0289	1	a
rF	Activity coefficient for F^-	0.315	1	a

Data source: a. Calculated using Geochemist's Workbench.

Supplementary Table 6. The characteristics of the sites for data compilation of P species.

Ocean	ODP sites	depth (m)	$CaCO_3$ (%)	sedimentation rate (m/m.y.)	Latitude (°)	Longitude (°)	bottom water temperature (°C)
Altlantic	1049	1345	34-92	3.6-12	30.14N	76.11W	
Altlantic	1050	1345	25-94	1.5-25	30.10N	76.24W	
Altlantic	1051	1345	30-90	18-36	30.05N	76.36W	3.7
Altlantic	1052	1345	11-91	18-26	29.95N	76.63W	
Indian	689	2080	61-99	2-10	64.52S	3.10E	1.0[a]
Indian	690	2914	73-93	5-50	65.17S	1.20E	
Indian	738	2253	84-96	1-31	62.72S	82.78E	
Pacific	1221	5175	0-64	1.3-18.8	12.03N	143.69W	
Pacific	844	3415	5-70	6-47	7.92N	90.48W	
Pacific	846	3296	30-70	10-61	3.10S	90.82W	1.7
Pacific	851	3760	70-80	16-55	2.77N	110.57W	1.4
Pacific	803	3410	83-94	8-30	2.43N	160.54E	
Pacific	804	3861	77-94	7-24	1.00N	161.59E	
Pacific	806	2520	90-97	19-43	0.32N	159.36E	2.0[b]
Atlantic	925	3041	35-84	10-50	4.2N	43.5W	
Atlantic	926	3598	48-81	11-29	3.7N	42.9W	1.0[a]
Atlantic	929	4356	5-71	3-37	6.0N	43.7W	

Note:

a. Data from Lear et al. [62].

b. Data from Lear et al. [63].

Supplementary Table 7. The environmental forcing values for the Phanerozoic look-up tables derived from our diagenetic model.

Time (My)	Ca[b] (mM)	Time (My)	DIC[a] (mM)	Time (My)	Mg[b] (mM)	Time (My)	pH[c]	Time (Myr)	xbt[d] (cm)	xbi[d] (cm)	Poro -sity[d]	Db[d] (cm^2 yr^{-1})	a0[d] (yr)
0.0	10.0	0.0	2.25	0.0	53.0	0.0	7.87	0.0	4.25	2.00	0.64	3.16	100
1.6	10.2	9.8	1.89	6.1	50.3	4.7	7.81	25.0	4.04	1.96	0.64	3.01	98.2
7.8	11.9	30.7	1.90	13.1	45.5	9.6	7.73	50.0	3.84	1.93	0.64	2.85	96.4
13.0	12.8	51.4	2.20	21.0	40.9	12.6	7.67	75.0	3.63	1.89	0.64	2.70	94.6
23.6	14.3	90.5	2.20	26.4	38.5	17.4	7.62	100.0	3.43	1.86	0.63	2.55	92.8
32.3	15.6	109.0	3.28	32.5	36.7	22.4	7.56	125.0	3.22	1.82	0.63	2.39	91.1
42.0	16.9	130.4	2.53	42.2	34.8	29.2	7.48	150.0	3.01	1.79	0.63	2.24	89.3
50.8	17.8	151.1	3.56	58.2	33.1	38.1	7.42	175.0	2.81	1.75	0.63	2.09	87.5
63.0	18.7	169.5	3.70	77.1	31.3	52.9	7.42	200.0	2.60	1.71	0.63	1.94	85.7
71.8	19.5	190.2	6.07	88.7	30.8	61.7	7.39	225.0	2.40	1.68	0.63	1.78	83.9
85.9	20.3	211.1	5.90	109.0	30.2	70.6	7.36	250.0	2.19	1.64	0.62	1.63	82.1
100.8	21.2	229.5	4.73	132.1	29.9	80.4	7.35	275.0	1.99	1.61	0.62	1.48	80.3
114.8	21.9	250.4	5.28	156.7	29.8	84.3	7.31	300.0	1.78	1.57	0.62	1.32	78.5
128.1	22.4	271.2	3.60	177.3	29.9	88.3	7.27	325.0	1.57	1.53	0.62	1.17	76.7
140.4	22.8	289.8	4.27	188.8	30.5	90.2	7.23	350.0	1.37	1.50	0.62	1.02	74.9
154.4	22.9	310.2	2.40	202.1	31.5	99.0	7.20	375.0	1.16	1.46	0.62	0.86	73.2
165.8	22.6	329.0	2.66	209.7	32.2	102.9	7.18	400.0	0.96	1.43	0.62	0.71	71.4
178.1	21.7	350.4	2.68	215.4	33.2	106.9	7.19	425.0	0.80	1.40	0.61	0.59	70.0
187.8	20.8	370.2	3.98	219.4	34.4	112.8	7.21	450.0	0.80	1.40	0.61	0.59	70.0
194.8	20.0	409.1	5.05	222.7	35.6	118.8	7.18	475.0	0.50	1.00	0.59	0.37	50.0
202.7	18.7	429.7	5.25	226.6	37.8	122.7	7.15	500.0	0.10	0.90	0.56	0.07	45.0
208.8	17.6	449.7	5.27	230.3	40.1	127.5	7.13	525.0	0.10	0.90	0.56	0.07	45.0
215.8	16.0	470.1	5.85	237.6	45.8	131.5	7.12	550.0	0.00	0.00	0.54	0.00	0.0
221.1	15.0	491.3	5.34	244.4	50.6	138.4	7.13						
225.5	14.0	509.3	5.86	244.4	50.6	144.3	7.15						
231.6	13.0	530.4	5.87	248.9	53.5	148.2	7.17						
236.0	12.4	548.6	8.09	254.3	55.4	155.1	7.17						
243.9	11.5			258.5	56.0	162.0	7.13						
253.6	11.1			264.2	56.2	169.8	7.09						
263.2	10.8			268.9	55.5	177.7	7.06						
271.2	10.8			273.2	54.6	187.5	7.09						
281.8	11.2			278.6	52.9	192.5	7.08						
298.4	11.9			284.2	50.3	199.3	7.09						
309.0	12.6			293.0	46.8	210.2	7.11						
316.0	13.4			300.5	43.7	216.1	7.15						
323.0	14.4			305.9	42.1	222.9	7.20						
329.1	15.8			312.1	40.8	225.9	7.22						

333.5	17.1	321.1	39.4	234.7	7.22
338.8	20.1	332.4	38.2	236.6	7.29
345.8	22.6	343.9	37.3	241.6	7.37
351.1	24.7	358.3	36.8	248.4	7.46
358.1	26.5	371.3	36.9	251.4	7.51
368.6	28.1	387.9	37.4	259.3	7.54
380.0	28.6	408.7	38.4	262.3	7.54
391.4	29.0	428.0	39.1	268.2	7.49
400.2	29.0	454.2	40.2	272.1	7.47
419.6	28.7	474.0	40.9	275.0	7.47
439.8	28.1	480.5	41.4	278.9	7.50
453.0	28.0	494.0	42.3	285.9	7.56
465.3	27.8	503.2	43.3	293.7	7.61
480.2	27.3	510.3	44.8	299.6	7.62
493.3	26.6	516.3	46.4	307.5	7.64
501.2	25.9	521.6	48.5	314.3	7.61
506.5	25.2	525.5	50.3	319.3	7.57
512.7	24.2	530.3	53.4	326.1	7.49
517.9	23.0	535.2	56.9	332.1	7.44
528.4	19.9	539.7	60.7	336.9	7.37
533.7	17.3	543.8	64.5	341.9	7.36
539.0	15.3	549.2	67.8	348.7	7.36
542.5	13.7			354.6	7.33
547.8	10.7			362.5	7.29
				369.4	7.23
				375.3	7.17
				383.2	7.13
				388.0	7.12
				396.9	7.11
				404.8	7.13
				408.7	7.18
				412.6	7.23
				416.5	7.24
				419.6	7.24
				427.4	7.17
				433.3	7.10
				441.2	7.09
				451.9	7.07
				457.8	7.07
				462.8	7.08
				468.7	7.08
				480.5	7.05
				484.4	7.05

492.2	7.08
500.1	7.12
506.0	7.14
511.9	7.17
517.8	7.19
524.7	7.20
535.5	7.20
551.3	7.18

Note:

a. Derived from ref. 35.
b. Derived from ref. 27.
c. Derived from ref. 36.
d. Evaluation based on results from ref. 37.

Supplementary Table 8. The parameters for the shallow-water and deep-sea diagenetic models for the Phanerozoic runs.

symbol	Shallow water	Deep sea	unit
T	12	3.5	^{o}C
S	35.5	35.5	1
P	2	328	bar
ω_0	0.2	0.0587	cm yr^{-1}
γ	0.08	0.08	1
a	0.15	50	yr
v	0.12	0.2	1
r_{P1}	See text	See text	
r_{P2}	See text	See text	
J.Fe(OH)$_3$$^{\alpha}$	6.85E-3	6.85E-4	mmol cm^{-2} yr^{-1}
J.Fe_3O_4	2E-3	0	mmol cm^{-2} yr^{-1}
J.biotite	4E-3	4E-4	mmol cm^{-2} yr^{-1}
J.$MnO_2$$^{\alpha}$	3.27E-3	3.27E-4	mmol cm^{-2} yr^{-1}
J.Calcite	0.432	0.0432	mmol cm^{-2} yr^{-1}
J.CFA	5.27E-5	5.27E-6	mmol cm^{-2} yr^{-1}
$[NO_3^-]_{BW}$	1.18E-5	3.16E-5	M
$[\Sigma PO_4^{3-}]_{BW}$	1E-6	2.4E-6	M
$[Na]_{BW}$	0.481	0.481	M

Note:

1. time-dependent parameters are shown in Table S6. For bioturbation of deep-sea sediments, xbi and xbt are the same as for shallow water, whereas D_{B0} and a_0 were scaled from shallow-water values by 0.32 and 0.1, respectively.

2. J_{OC} and $[O_2]_{BW}$ are coupled with the carbon cycle model.

3. All other parameters are the same as at the FOAM site.

Supplementary Table 9. Flux equations and reservoir equations for the coupled carbon-phosphorus-oxygen cycle model.

Flux equations

$F_{54} = k_{5-4} \cdot v_{mix} \cdot M_5$	(C1)
$F_{13} = CP_{Redfield} \cdot F_{54}$	(C2)
$F_{36} = k_{3-6} \cdot F_{13}^{2.5}$	(C3)
$F_{31} = F_{13} - F_{36}$	(C4)
$F_{47} = \frac{F_{36}}{CP_{burial}}$	(C5)
$F_{58} =$ output from the diagenetic model	(C6)
$F_{59} = k_{5\_9} * F_{1112}$	(C7)
$F_{45} = F_{54} - F_{47}$	(C8)
$F_{25} = k_{2-5} \cdot M_2$	(C9)
$F_{61} = k_{uplift} \cdot M_6$	(C10)
$F_{72} = k_{uplift} \cdot M_7$	(C11)
$F_{82} = k_{uplift} \cdot M_8$	(C12)
$F_{92} = k_{uplift} \cdot M_9$	(C13)
$F_{1113} = x \cdot F_{1011}$	(C14)

$F_{1112} = (1 - x) \cdot F_{1011}$	(C15)
$F_{1210} = k_{uplift} \cdot M_{12}$	(C16)
$F_{1310} = k_{uplift} \cdot M_{13}$	(C17)
$F_{1011} = k_{10\_11} \cdot M_{10}$	(C18)

Reservoir equations

$\frac{dM_1}{dt} = F_{31} + F_{61} - F_{13}$	(C19)
$\frac{dM_2}{dt} = F_{72} + F_{82} + F_{92} - F_{25}$	(C20)
$\frac{dM_3}{dt} = F_{13} - F_{31} - F_{36}$	(C21)
$\frac{dM_4}{dt} = F_{54} - F_{45} - F_{47}$	(C22)
$\frac{dM_5}{dt} = F_{25} + F_{45} - F_{54} - F_{58} - F_{59}$	(C23)
$\frac{dM_6}{dt} = F_{36} - F_{61}$	(C24)
$\frac{dM_7}{dt} = F_{47} - F_{72}$	(C25)
$\frac{dM_8}{dt} = F_{58} - F_{82}$	(C26)

$\frac{dM_9}{dt} = F_{59} - F_{92}$	(C27)
$\frac{dM_{10}}{dt} = F_{1310} + F_{1210} - F_{1011}$	(C28)
$\frac{dM_{11}}{dt} = F_{1011} - F_{1112} - F_{1113}$	(C29)
$\frac{dM_{12}}{dt} = F_{1112} - F_{1210}$	(C30)
$\frac{dM_{13}}{dt} = F_{1113} - F_{1310}$	(C31)
$\frac{dM_{14}}{dt} = F_{13} - (F_{31} - 3.5F_{1113}) - 3.5F_{1310} - F_{61}$	(C32)

Other equations

$DOA = 1 - k_{OA}(\frac{v_{mix}M_{14}}{F_{13}})$	(C33)
$x = 0.44 + 0.56(DOA)$	(C34)
$(\frac{C}{P})_{burial} = \frac{(C/P)_{oxic}(C/P)_{anoxic}}{(1 - DOA)(C/P)_{anoxic} + DOA(C/P)_{oxic}}$	(C35)

Supplementary Table 10. Pre-industrial steady-state reservoirs of the coupled carbon-phosphorus-oxygen exogenic cycles.

Reservoirs	Description	Value (mol)	Reference
M1	Total mass oxidized carbon in exogenic cycle + terrestrial organic carbon + terrestrial organic carbon + oceanic dissolved organic carbon	6.50E+21	ref. 64
M2	Total exogenic phosphorus minus other P reservoirs	2.50E+19	This study
M3	Particulate organic carbon in oceans	3.50E+15	ref. 64
M4	Particulate organic phosphorus in oceans	1.80E+13	ref. 64
M5	Total marine P	2.00E+15	ref. 64
M6	Marine sedimentary organic carbon	1.30E+21	ref. 64
M7	Marine sedimentary organic phosphorus	1.0E+19	This study
M8	Phosphate in authigenic calcium phosphate mineral, biogenic apatite and biogenic $CaCO_3$	1.0E+19	This study
M9	Phosphate absorbed to non-detrital ferric (hydro)oxides (volcanogenic + authigenic)	2.0E+18	ref. 64
M10	Terrestrial iron and unreactive marine iron	1.3E+21	ref. 65
M11	Reactive Fe (III) in the ocean	1	ref. 65
M12	Reactive Fe (III) in sediments	1.2E+20	ref. 65
M13	Pyrite in sediments	1.5E+20	ref. 65
M14	Ocean + atmosphere O_2	3.80E+19	ref. 65

Supplementary Table 11. Pre-industrial steady-state fluxes in the coupled carbon-phosphorus-oxygen exogenic cycles.

Fluxes	Description	Value (mol/yr)	Reference
F13	Net primary production in oceans	2.50E+15	ref. 64
F54	Biological fixation of reactive phosphorus (F13/106)	2.36E+13	ref. 64
F36	Marine sedimentary burial of organic carbon	3.75E+12	ref. 64
F31	Respiration and decomposition in the oceans (F13-F36)	2.50E+15	ref. 64
F47	Marine sedimentary burial of organic phosphorus (F36/250)	2.90E+10	This study
F45	Oceanic recycling of particulate organic phosphorus (F54-F47)	2.36E+13	ref. 64
F58	Marine sedimentary burial of mineral calcium-bound phosphate	2.90E+10	This study
F59	Marine sedimentary burial of phosphate sorbed to reactive ferric (hydro)oxides	6.00E+09	ref. 64
F25	Reactive phosphorus flux from continents to oceans	6.40E+10	This study
F61	Uplift and exposure of marine sedimentary organic carbon (= F36)	3.75E+12	ref. 64
F72	Uplift and exposure of marine sedimentary organic phosphorus (= F47)	2.90E+10	This study
F82	Uplift and exposure of marine sedimentary mineral calcium-bound phosphate (= F58)	2.90E+10	This study
F92	Uplift and exposure of marine sedimentary phosphate sorbed to reactive ferric (hydro)oxides (= F59)	6.00E+09	ref. 64
F1113	Marine sedimentary burial of pyrite	4.4E+11	ref. 65
F1112	Marine sedimentary burial of reactive Fe (III)	3.5E+11	ref. 65
F1210	Uplift and exposure of marine sedimentary reactive Fe (III)	3.5E+11	ref. 65
F1310	Uplift and exposure of marine sedimentary pyrite	4.4E+11	ref. 65
F1011	Reactive Fe (III) flux to the oceans	7.9E+11	ref. 65

Supplementary Table 12. The value of parameters for the coupled carbon-phosphorus-oxygen cycle model.

Model parameters	Value	Unit	Reference
v_{mix}	3	m yr^{-1}	ref. 64
n	2.5		ref. 64
$CP_{Redfield}$	106		ref. 64
CP_{burial}	250		ref. 64
k_{5-4}	3.93E-03	m^{-1}	ref. 64
k_{3-6}	1.20E-26	$(mol/yr)^{-1.5}$	ref. 64
$(C/P)_{oxic}$	106		ref. 17
$(C/P)_{anoxic}$	500		ref. 17
K_{5_9}	1.71E-20	mol yr^{-1}	ref. 65
k_{OA}	1.73E-5	mol m^{-1}	ref. 65
k_{2-5}	2.57E-09	yr^{-1}	ref. 64
K_{10-11}	6.08E-10	yr^{-1}	ref. 65
k_{uplift}	2.88E-09	yr^{-1}	ref. 64

Supplementary Table 13. Plant evolution parameters for the coupled carbon-phosphorus-oxygen cycle model.

Age (yr)	Plant evolution
-1.00E+09	0
-6.00E+08	0
-4.65E+08	0
-4.45E+08	0.15
-4.00E+08	0.15
-3.50E+08	1
1.00E+09	1

Supplementary Figures

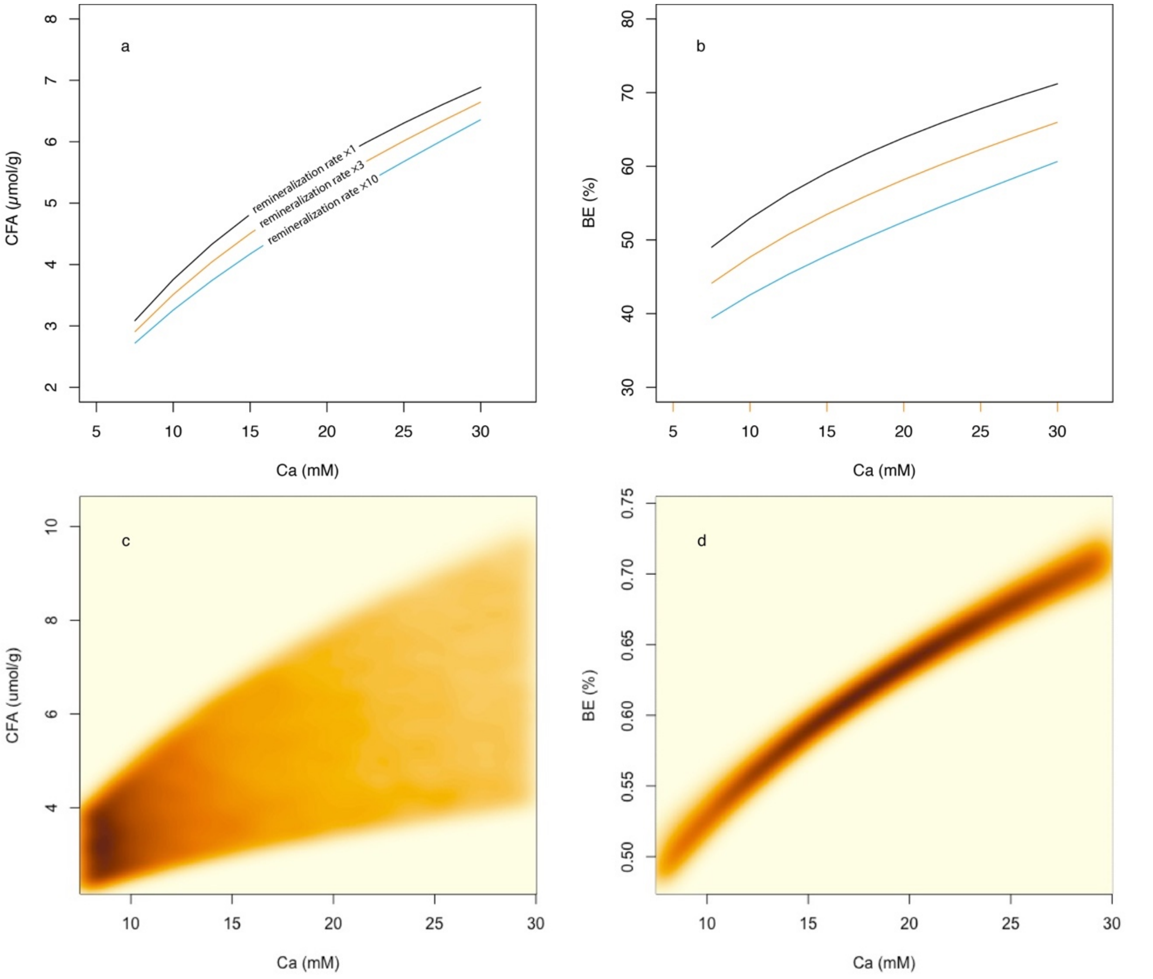

Supplementary Figure 1. The influence of seawater Ca concentration on CFA burial and P burial efficiency, as derived from our basic diagenetic model. a.b. runs with varying remineralization rate of organic matter (1, 3 and 10 times of the baseline run). **c.d.** a Monte carlo simulation with 200000 runs. Parameters (J_{OC}, $[DIC]_{BW}$, $[PO4]_{BW}$) are sampled using uniform distribution. The ranges of parameters are 1.5 to 4.5 mol m^{-2} yr^{-1} for J_{OC}, 2 to 8 mM for $[DIC]_{BW}$ and 1 to 3 μM for $[PO4]_{BW}$.

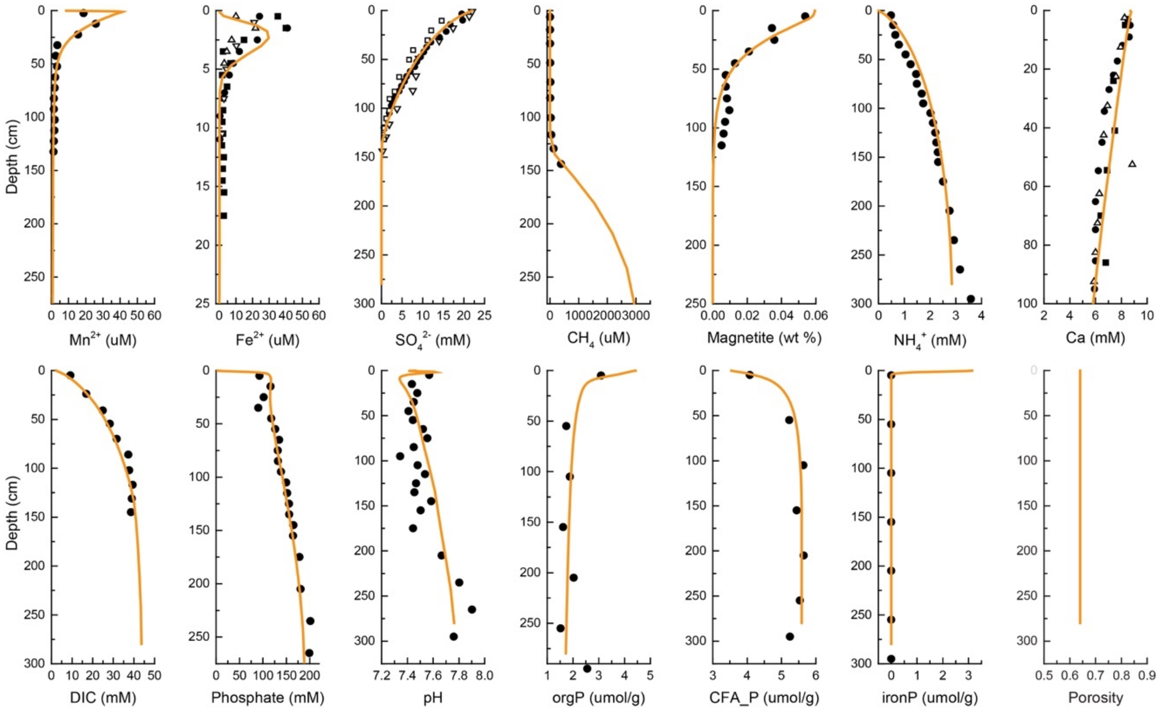

Supplementary Figure 2. The application of the diagenetic model to the FOAM site. Data are from refs. 1-9. Curves represent the model output.

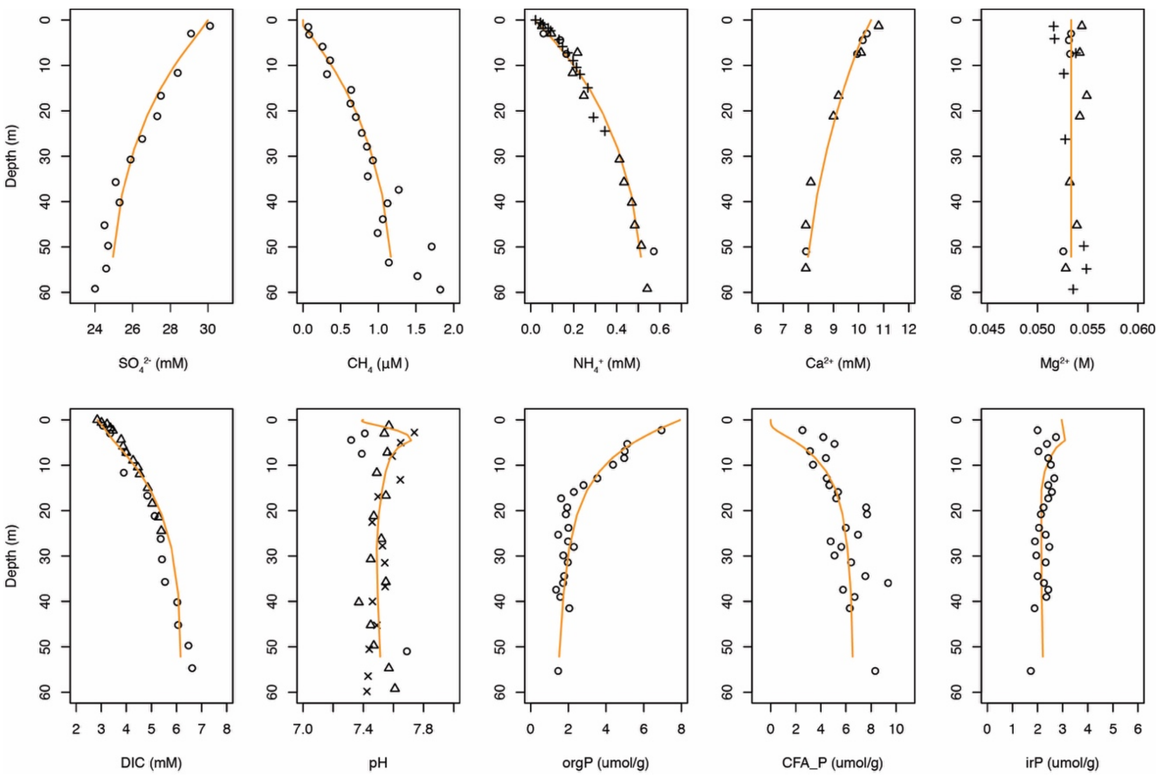

Supplementary Figure 3. The application of the diagenetic model to ODP site 846/1226. Data are from refs. 23-26. Curves represent the model output.

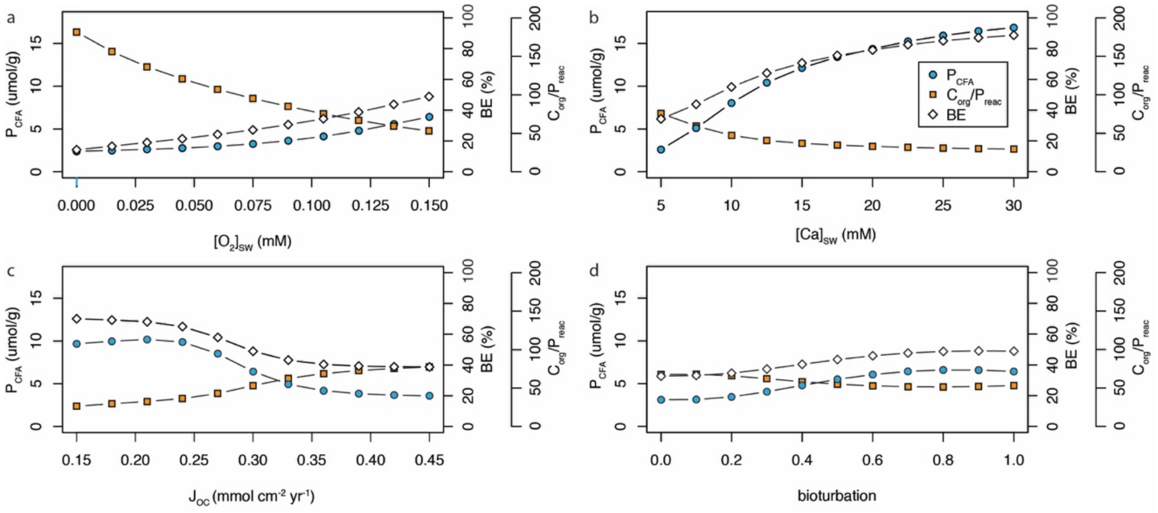

Supplementary Figure 4. Nonlinear effects of environmental factors on CFA formation, total P burial efficiency and C_{org}/P_{reac}. In compare with Figure 2, organic C/P ratios are parametrized as a function of seawater oxygen level using Equations (15) and (16) of the main text. P_{CFA} (circles) represents burial concentration of CFA-associated phosphorus in marine sediments. BE (diamonds) represents the burial efficiency of reactive phosphorus in marine sediments. C_{org}/P_{reac} represents the burial ratio between organic carbon and reactive P. **a-d**. The effects upon P_{CFA}, BE and C_{org}/P_{reac} of bottom-water oxygen concentration (a), seawater dissolved calcium concentration (b), flux of organic carbon to the sediment-seawater interface (J_{oc}) (c) and bioturbation (d). For bioturbation, the four parameters included in the model (DB_0, a_0, xbt and xbi) were increased linearly from zero to modern average values, while a linear increase of 16% was applied to porosity. Bioturbation is parameterized here as a coupled biodiffusion and bioirrigation term.

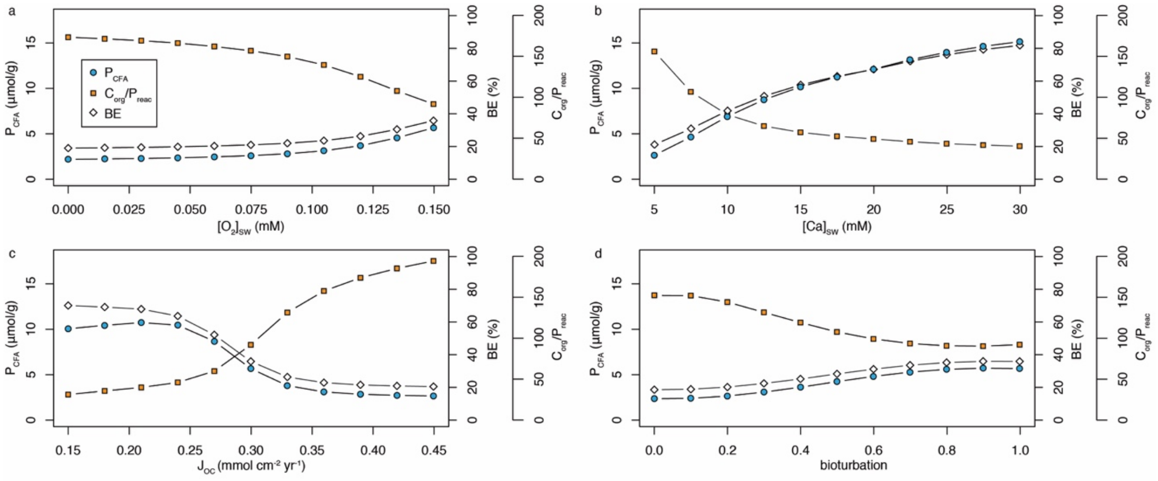

Supplementary Figure 5. Nonlinear effects of environmental factors on CFA formation, total P burial efficiency and C_{org}/P_{reac}. In compare with Figure 2, K_{spCFA} is fixed as $10^{-99.7}$ without a relationship with carbonate activity, and k26 = 2.7×10^{-12} M yr^{-1}. P_{CFA} (circles) represents burial concentration of CFA-associated phosphorus in marine sediments. BE (diamonds) represents the burial efficiency of reactive phosphorus in marine sediments. C_{org}/P_{reac} represents the burial ratio between organic carbon and reactive P. **a-d**. The effects upon P_{CFA}, BE and C_{org}/P_{reac} of bottom-water oxygen concentration (a), seawater dissolved calcium concentration (b), flux of organic carbon to the sediment-seawater interface (J_{oc}) (c) and bioturbation (d). For bioturbation, the four parameters included in the model (DB_0, a_0, xbt and xbi) were increased linearly from zero to modern average values, while a linear increase of 16% was applied to porosity. Bioturbation is parameterized here as a coupled biodiffusion and bioirrigation term.

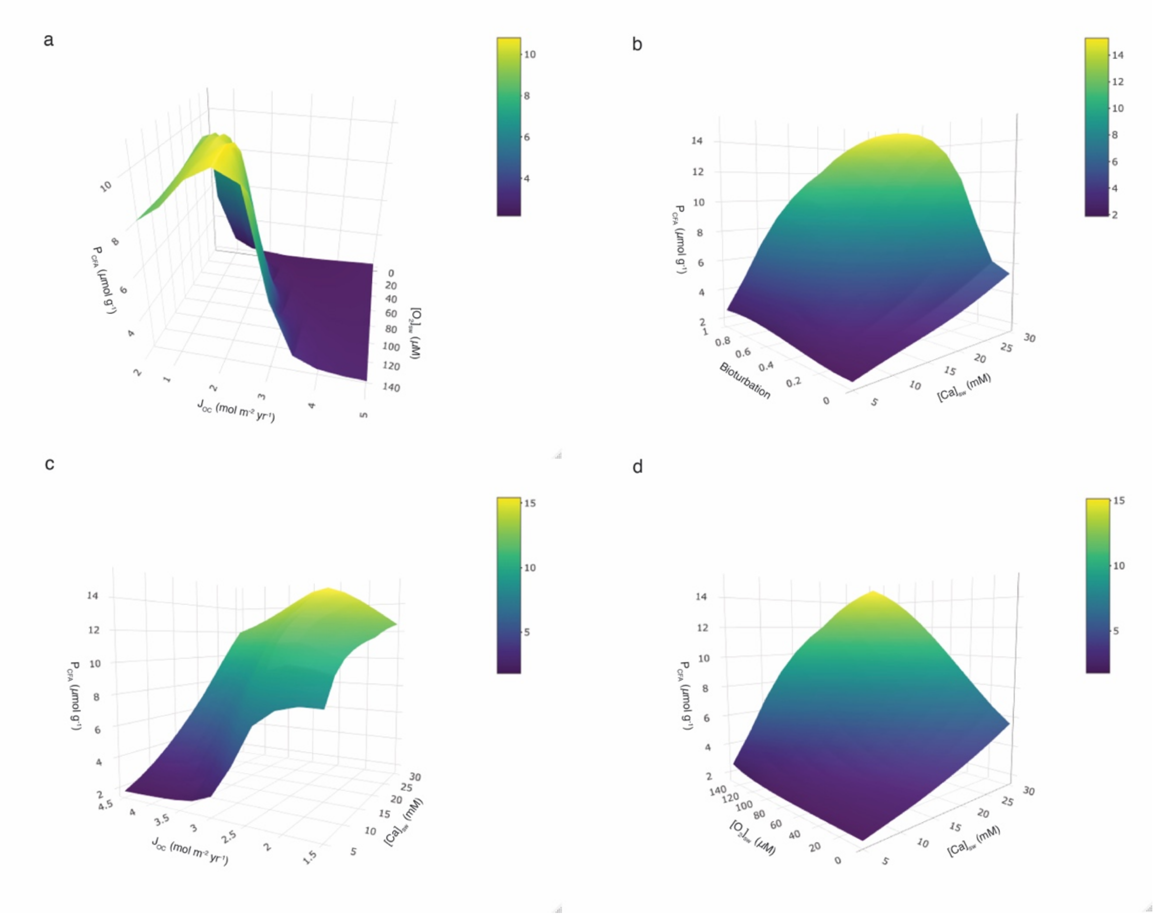

Supplementary Figure 6. Nonlinear effects of environmental factors on CFA formation. (a). Flux of organic carbon to the sediment-seawater interface (J_{oc}) and bottom-water oxygen concentration. (b). Seawater dissolved calcium concentration and bioturbation. (c). Seawater dissolved calcium concentration and flux of organic carbon to the sediment-seawater interface (J_{oc}). (d). Seawater dissolved calcium concentration and bottom-water oxygen concentration. For bioturbation, the four parameters included in the model (DB_0, a_0, xbt and xbi) were increased linearly from zero to modern average values, while a linear increase of 16% was applied to porosity. Bioturbation is parameterized here as a coupled biodiffusion and bioirrigation term. In these runs, K_{spCFA} is fixed as $10^{-99.7}$ without a relationship with carbonate activity, and k26 = 2.7×10^{-12} M yr^{-1}.

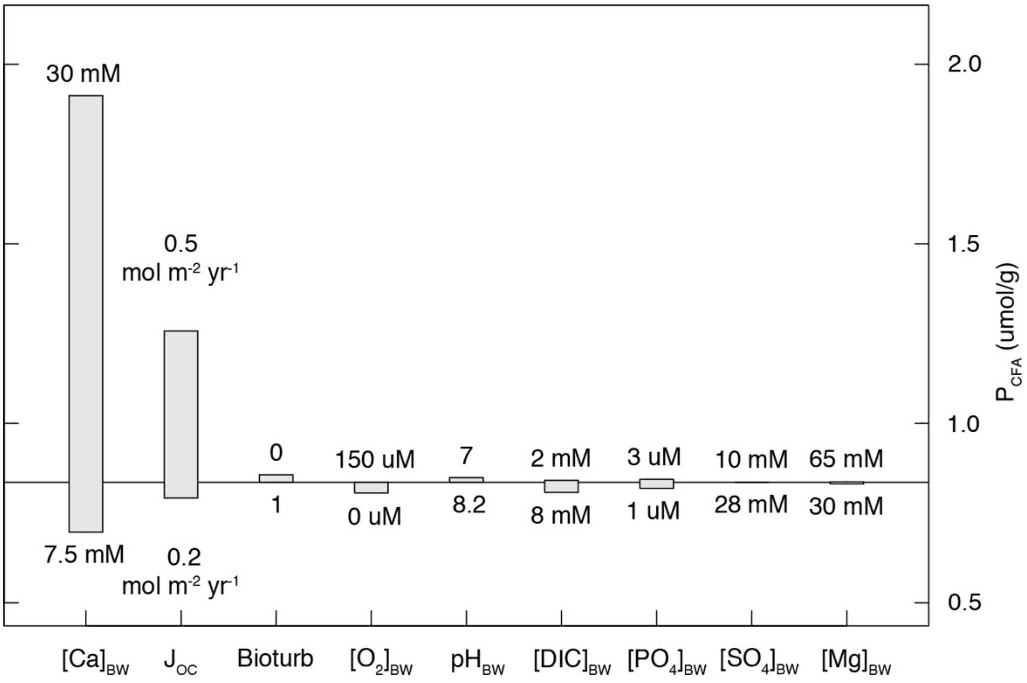

Supplementary Figure 7. Environmental forcings on the burial of CFA in deep-sea settings. The y-axis is the CFA burial concentration of P in sediments. The line represents the value for the reference model run. Other than the parameter for each sensitivity analysis, the other parameters were held constant for each run.

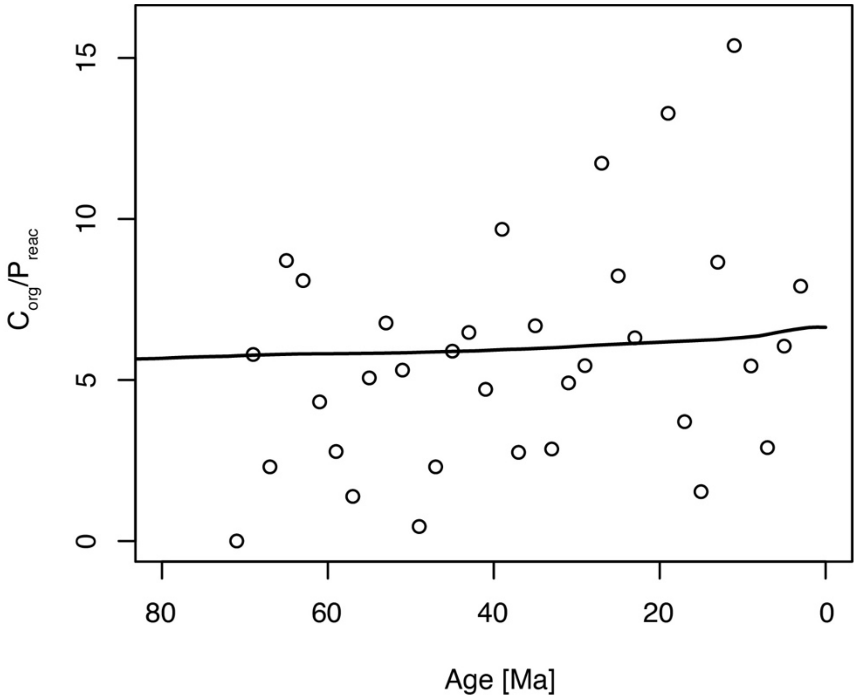

Supplementary Figure 8. Deep sea records of C_{org}/P_{reac} over the past 80 million years. The data of reactive P (P_{reac}) are the same with the P_{CFA}/P_{reac} compilation (Fig. 4), while the data of organic carbon comes from the ODP database (http://web.iodp.tamu.edu/OVERVIEW/?&set=1). The black line represents the result from the same model run in Fig. 4, assuming 5% of organic carbon burial[66] and 50% of reactive P burial occur in deep sea[9]. Points represent the ratio between the mean values of C_{org} and the mean value of P_{reac} for each 2 myr bin. Note that the C_{org} and P_{reac} are not the results of the same samples.

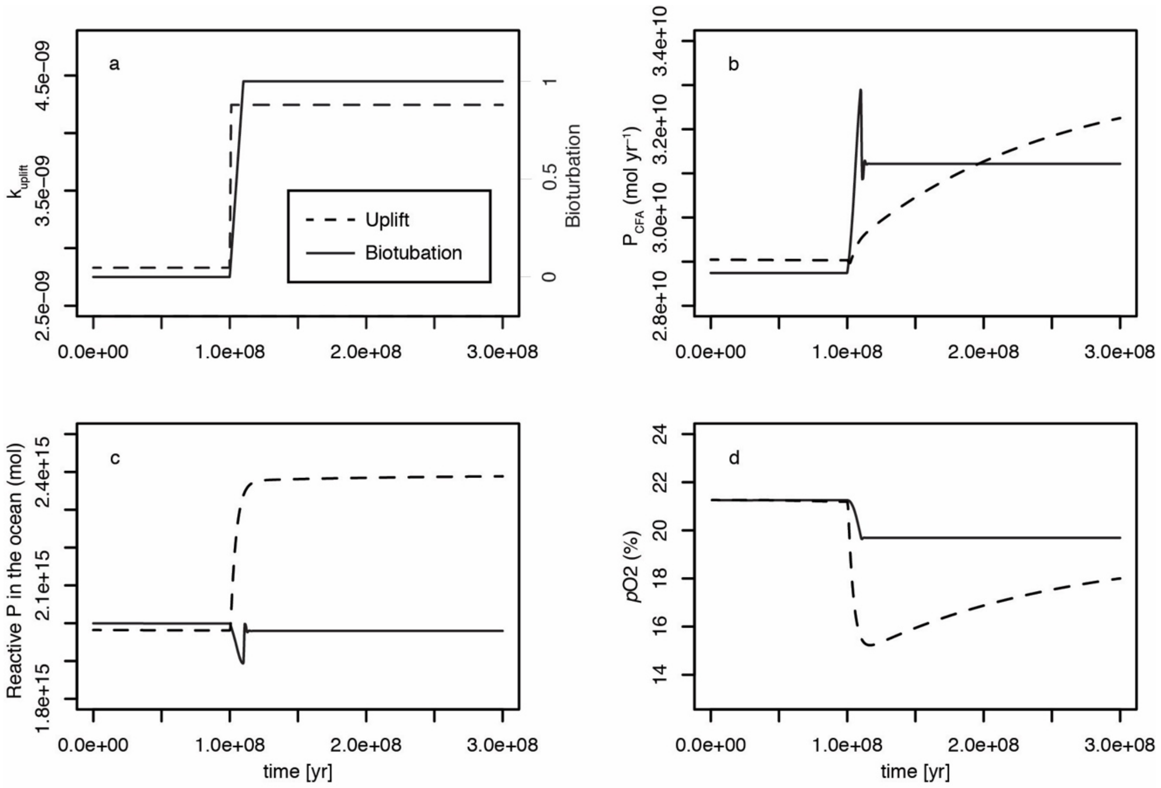

Supplementary Figure 9. The response of atmospheric oxygen levels to changes in uplift and bioturbation. a. Model input of uplift or bioturbation ("0" represents no bioturbation, while "1" represents 'modern' (FOAM baseline) levels of bioturbation). **b,c,d.** model outputs of variations in P burial associated with CFA formation, total marine P (a sum of dissolved inorganic phosphate, dissolved organic phosphorus and soluble particulate inorganic phosphorus) in the ocean water and atmospheric oxygen levels.

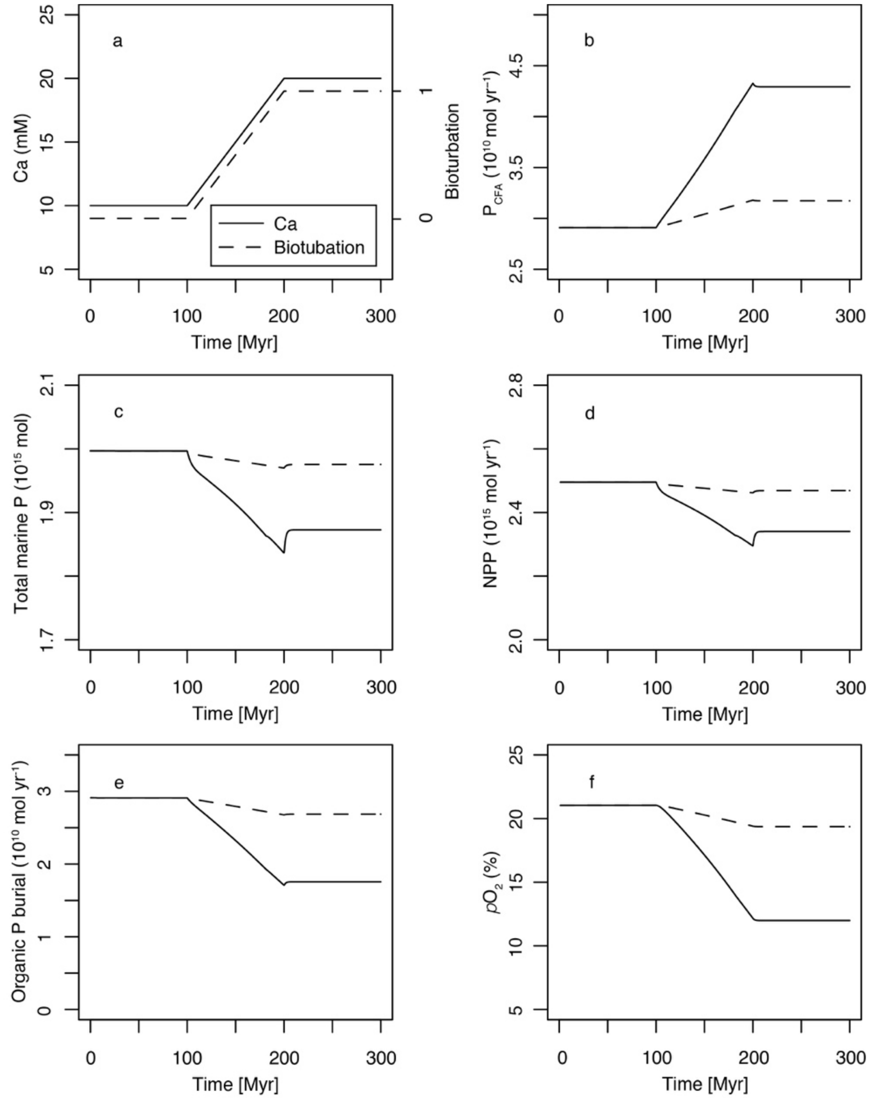

Supplementary Figure 10. The response of atmospheric oxygen levels to changes in seawater dissolved Ca concentration or bioturbation. In compare with Figure 5, K_{spCFA} is fixed as $10^{-99.7}$ without a relationship with carbonate activity, and k26 = 2.7×10^{-12} M yr^{-1}. **a.** Model input of seawater dissolved Ca concentration or bioturbation ("0" represents no bioturbation, while "1" represents the reach of modern bioturbation). **b,c,d,e,f.** model outputs of variations in P burial associated with CFA formation, total marine P (a sum of dissolved inorganic phosphate, dissolved organic phosphorus and soluble particulate inorganic phosphorus), net primary productivity (NPP), organic P burial flux and atmospheric oxygen levels. pO_2 shown in the figure is the actual percent by volume.

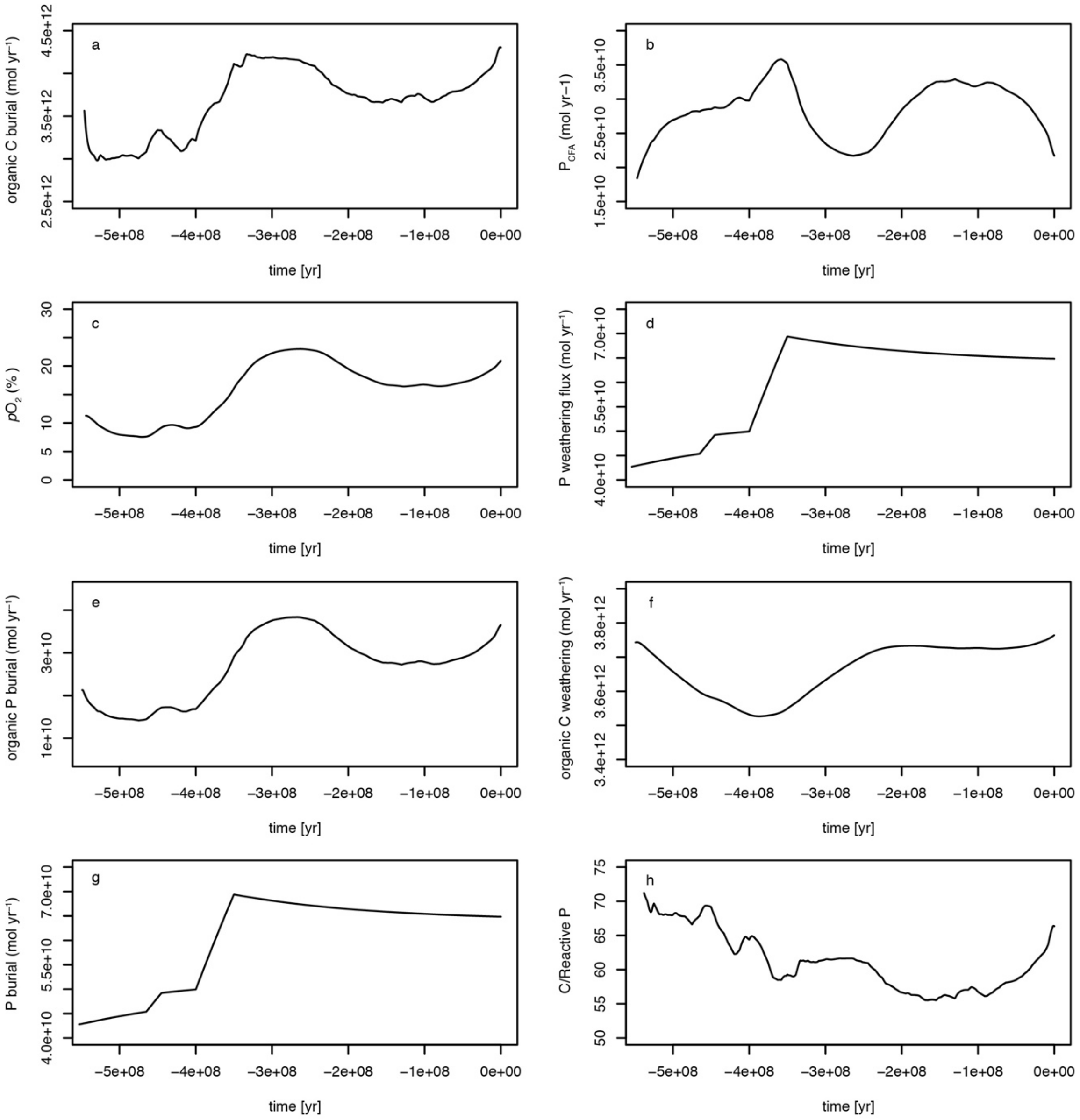

Supplementary Figure 11. Default output from the coupled carbon-phosphorus-oxygen cycle model. a. sedimentary burial flux of organic carbon; **b.** marine sedimentary burial flux of phosphorus in CFA; **c.** evolution of atmospheric O_2 levels; **d.** the weathering flux of phosphorus from land; **e.** marine sedimentary burial flux of organic phosphorus; **f.** uplift, exposure and weathering flux of marine sedimentary organic carbon; **g.** the burial flux of P. A plant-assisted weathering effect was included in this run.

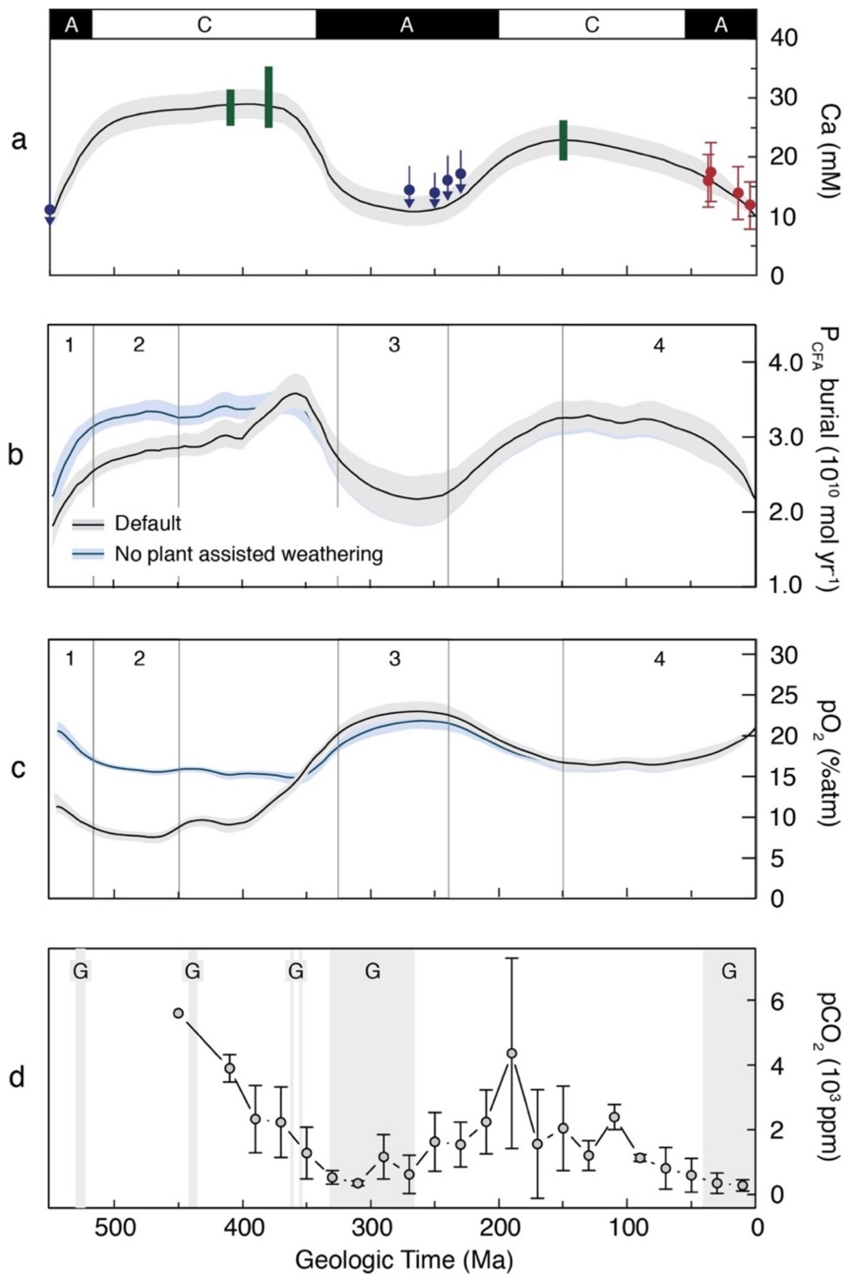

Supplementary Figure 12. A Phanerozoic Ca-mediated phosphorus-carbon-oxygen cycle. a. The Phanerozoic evolution of seawater dissolved calcium concentrations, using data from Horita et al.[32]. "A" and "C" represent intervals dominated by seawater precipitation of aragonite and calcite, respectively. **b**. Model results for Phanerozoic CFA-associated phosphorus burial (this study). **c**. Model results for the Phanerozoic evolution of atmospheric pO_2 (this study). **d**. Atmospheric pCO_2 as recorded by various proxy data compiled by Royer et al.[67]. Points represent mean values for 20 myr bins, with the error bars represent one standard deviation (1σ). The shaded intervals denoted as "G" represent intervals of glaciation. Sensitivity analyses were done to exploring the influence of plant-assisted weathering.

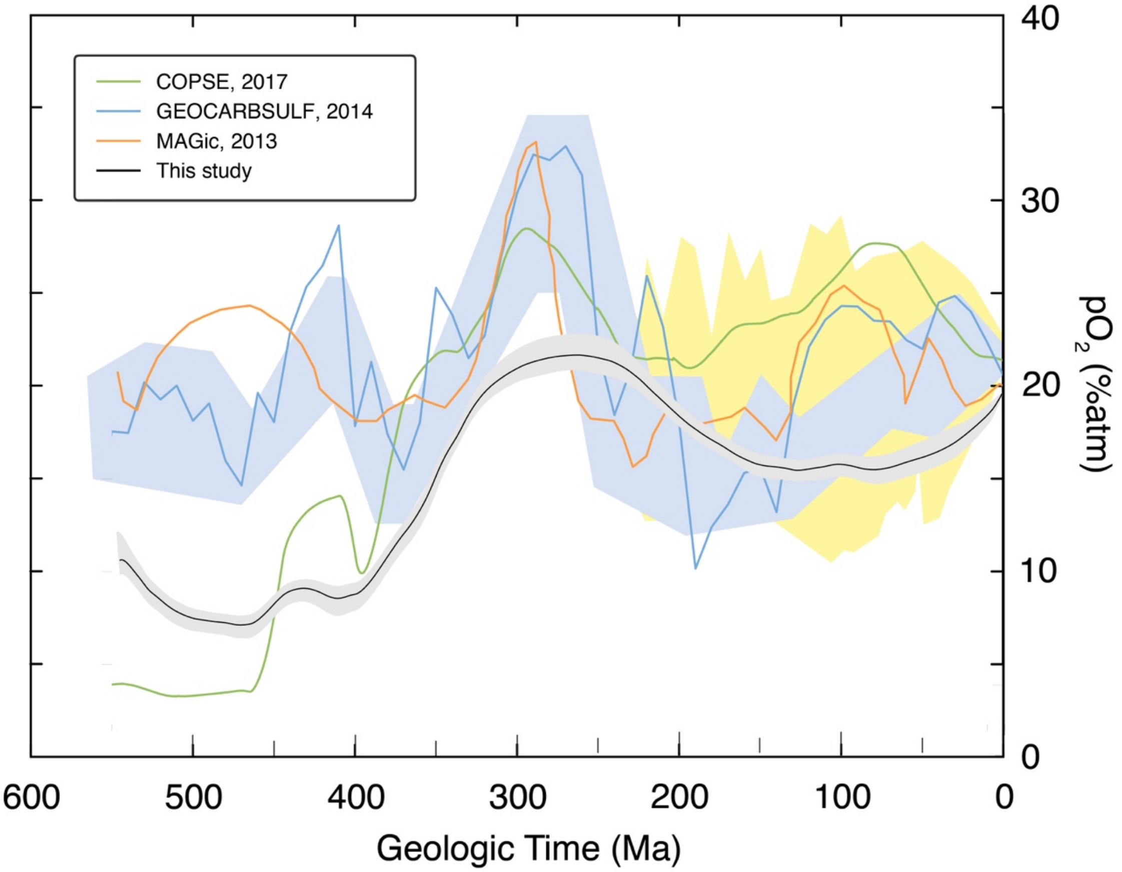

Supplementary Figure 13. Atmospheric O_2 results from different global models. Our model outputs, with incorporation of plant-assisted weathering (ap=0.5) and low marine dissolved Ca concentration during the Carboniferous–Permian, are represented by the black curve. The blue shadow represent GEOCARBSULF 2007 results of ref. 41. The yellow shadow represents the range of proxy results for the Mesozoic and Cenozoic compiled by ref. 46. The newest COPSE baseline model is the green curve[38]. The GEOCARBSULF 2014 model is the blue curve[67]. The MAGic model is the orange curve[68].

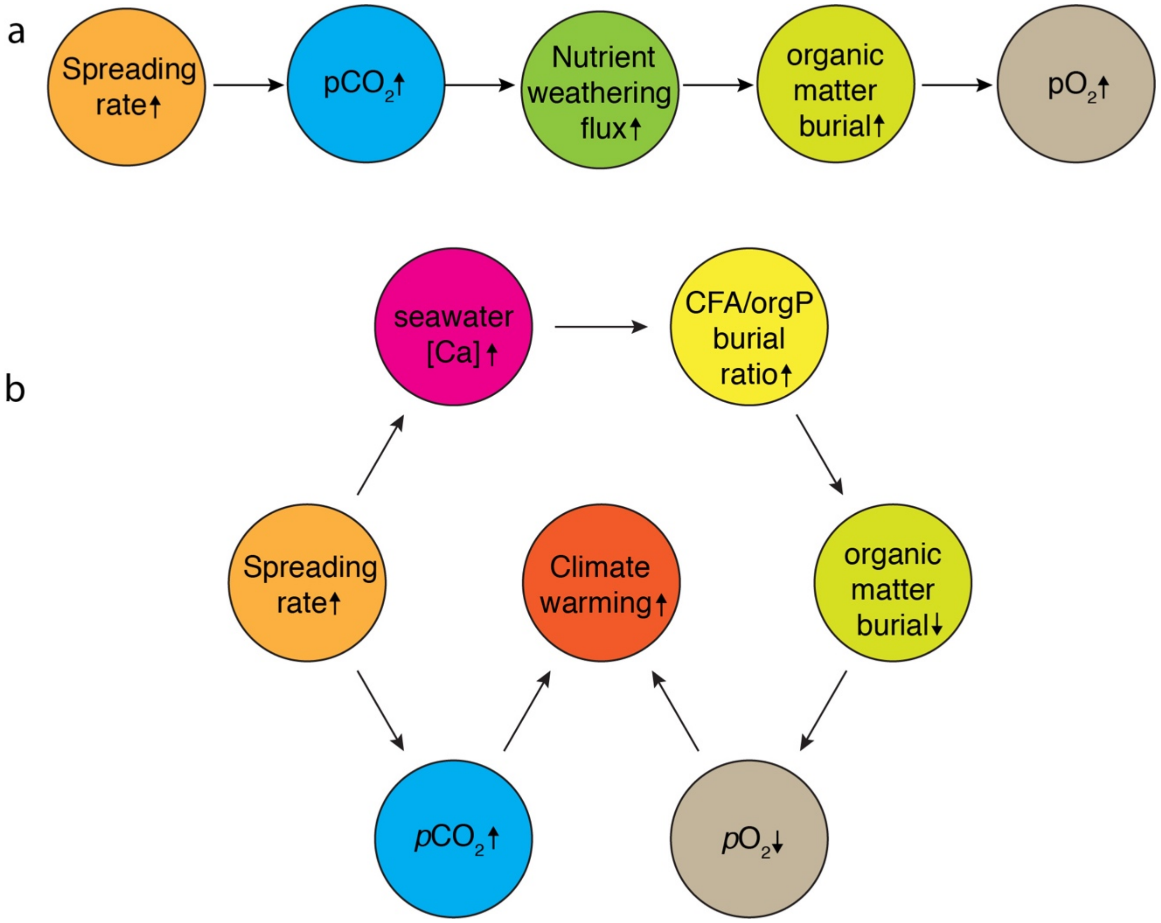

Supplementary Figure 14. The influence of tectonic activity on atmospheric *p*O_2 and climate. a. The traditional paradigm, derived from ref. 69; b. The framework proposed in this study.

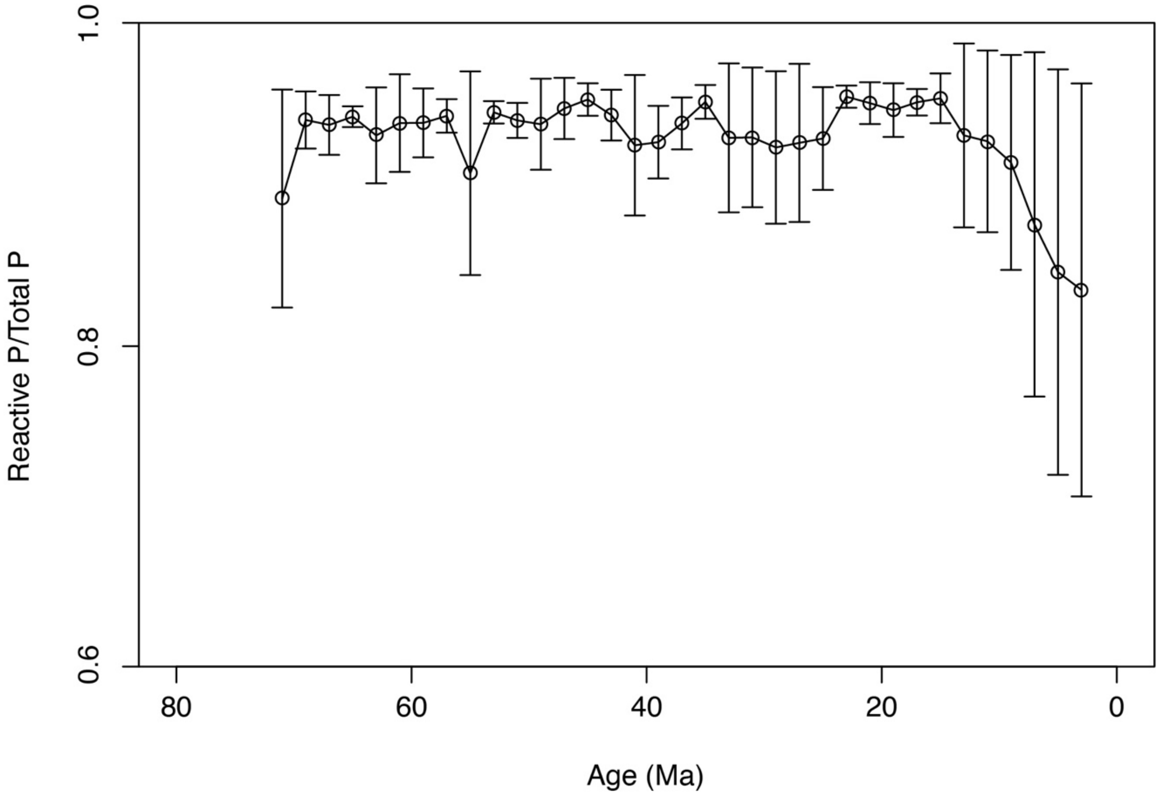

Supplementary Figure 15. Marine records of the variation of in the ratio of reactive P to total P in deep sea sediments over the past 80 million years. The data are from the same source as those shown in Fig. 4. The error bars represent one standard deviation (1σ).

Supplementary References:

1. Canfield, D. E. & Berner, R. A. Dissolution and Pyritization of Magnetite in Anoxic Marine-Sediments. *Geochim Cosmochim Ac* **51**, 645-659 (1987).
2. Krom, M. D. & Berner, R. A. The Diagenesis of Phosphorus in a Nearshore Marine Sediment. *Geochim Cosmochim Ac* **45**, 207-216 (1981).
3. Aller, R. C. Influence of macrobenthos on chemical diagenesis of marine sediments. Ph.D. dissertation, Yale Univ. (1977).
4. Goldhaber, M. B. *et al.* Sulfate Reduction, Diffusion, and Bioturbation in Long-Island Sound Sediments - Report of Foam Group. *Am J Sci* **277**, 193-237 (1977).
5. Martens, C. S. & Berner, R. A. Interstitial Water Chemistry of Anoxic Long Island Sound Sediments .1. Dissolved-Gases. *Limnol Oceanogr* **22**, 10-25 (1977).
6. Berner, R. A., Westrich, J. T., Graber, R., Smith, J. & Martens, C. S. Inhibition of aragonite precipitation from supersaturated seawater: a laboratory and field study. *Am J Sci* **278**, 816-837 (1978).
7. Canfield, D. E. Sulfate reduction and the diagenesis of iron in anoxic marine sediments. Ph.D. dissertation, Yale University (1988).
8. Ruttenberg, K. C., Diagenesis and burial of phosphorus in marine sediments: Implications for the marine phosphorus budget. Ph.D. dissertation, Yale Univ. (1990).
9. Ruttenberg, K. C. Reassessment of the Oceanic Residence Time of Phosphorus. *Chem Geol* **107**, 405-409 (1993).
10. R development core team, R: A language and environment for statistical computing. R Foundation for Statistical Computing 61, 1673-1676 (2006).
11. Soetaert K. & Meysman F. J. R. Reactive transport in aquatic ecosystems: Rapid model prototyping in the open source software R. *Environmental Modelling & Software* **32**, 49-60 (2012).
12. Soetaert K., Meysman F. J. R. & Petzoldt T. Solving Differential Equations in R. *AIP Conf. Proc. 1281, 31. DOI: 10.1063/1.3498463* (2010).
13. Ruttenberg, K. C. Development of a Sequential Extraction Method for Different Forms of Phosphorus in Marine-Sediments. *Limnol Oceanogr* **37**, 1460-1482 (1992).

14. Gunnars, A., Blomqvist, S. & Martinsson, C. Inorganic formation of apatite in brackish seawater from the Baltic Sea: an experimental approach. *Mar Chem* **91**, 15-26 (2004).
15. Aller, R. C., Diagenetic processes near the sediment-water interface of Long Island Sound. I. decomposition and nutrient element geochemistry (S, N, P). Adv Geophys **22**, 237-350 (1980).
16. Kim, D., Schuffert, J. D. & Kastner, M. Francolite authigenesis in California continental slope sediments and its implications for the marine P cycle. *Geochim Cosmochim Ac* **63**, 3477-3485 (1999).
17. Dale, A. W., Boyle, R. A., Lenton, T. M., Ingall, E. D. & Wallmann, K. A model for microbial phosphorus cycling in bioturbated marine sediments: Significance for phosphorus burial in the early Paleozoic. *Geochim Cosmochim Ac* **189**, 251-268 (2016).
18. Van Cappellen, P. & Berner, R. A. A Mathematical-Model for the Early Diagenesis of Phosphorus and Fluorine in Marine-Sediments - Apatite Precipitation. *Am J Sci* **288**, 289-333 (1988).
19. Slomp, C. P., Epping, E. H. G., Helder, W. & VanRaaphorst, W. A key role for iron-bound phosphorus in authigenic apatite formation in North Atlantic continental platform sediments. *J Mar Res* **54**, 1179-1205 (1996).
20. Kraal, P., Slomp, C. P., Reed, D. C., Reichart, G. J. & Poulton, S. W. Sedimentary phosphorus and iron cycling in and below the oxygen minimum zone of the northern Arabian Sea. *Biogeosciences* **9**, 2603-2624 (2012).
21. Koutsoukos, P., Amjad, Z., Tomson, M. B. & Nancollas, G. H. Crystallization of calcium phosphates. A constant composition study. J Am Chem Soc, **102**, 1553-1557 (1980).
22. Van Cappellen, P. & Berner, R. A. Fluorapatite Crystal-Growth from Modified Seawater Solutions. *Geochim Cosmochim Ac* **55**, 1219-1234 (1991).
23. Shipboard Scientific Party, Site 846. In *Proc. ODP, Init. Repts., vol. 138 (eds. L. Mayer, N. Pisias, T. Janecek, et al.)*. Ocean Drilling Program, College Station, TX, USA, 1992.
24. Shipboard Scientific Party, Site 1226. *In Proc. ODP, Init. Repts., vol. 201 [CD-ROM] (eds. S. L. D'Hondt, B. B. Jørgensen, D. J. Miller, et al.).* Ocean Drilling

Program, College Station, TX, USA, 2003.
25. Filippelli, G. M. & Delaney, M. L. Phosphorus geochemistry of equatorial Pacific sediments. *Geochim Cosmochim Ac* **60**, 1479-1495 (1996).
26. Wang GZ, Spivack AJ, D'Hondt S. Gibbs energies of reaction and microbial mutualism in anaerobic deep subseafloor sediments of ODP Site 1226. *Geochim Cosmochim Ac* **74**, 3938-3947 (2010).
27. Delaney, M. L. & Anderson, L. D., Phosphorus geochemistry in Ceara Rise sediments. *Proc. ODP, Sci. Results* **154**, 475-482 (1997).
28. Faul, K. L. & Delaney, M. L. Data report: Phosphorus concentrations and geochemistry in Blake Nose sediments from Leg 171B. *Proc. ODP, Sci. Results B* **171**, 1-10 (2000).
29. Faul, K. L. & Delaney, M. L. A comparison of early Paleogene export productivity and organic carbon burial flux for Maud Rise, Weddell Sea, and Kerguelen Plateau, south Indian Ocean. *Paleoceanography* **25**, PA3214 (2010).
30. Faul, K. L. & Paytan, A. Phosphorus and barite concentrations and geochemistry in Site 1221 Paleocene/Eocene boundary sediments. *Proc. ODP, Sci. Results* **199**, 1-23 (2005).
31. Kraal P, Slomp CP, Forster A, Kuypers MMM, Sluijs A. Pyrite oxidation during sample storage determines phosphorus fractionation in carbonate-poor anoxic sediments. *Geochim Cosmochim Ac* **73**, 3277-3290 (2009).
32. Horita, J., Zimmermann, H. & Holland, H. D. Chemical evolution of seawater during the Phanerozoic: Implications from the record of marine evaporites. *Geochim Cosmochim Ac* **66**, 3733-3756 (2002).
33. Coggon, R. M., Teagle, D. A. H., Smith-Duque, C. E., Alt, J. C. & Cooper, M. J. Reconstructing Past Seawater Mg/Ca and Sr/Ca from Mid-Ocean Ridge Flank Calcium Carbonate Veins. *Science* **327**, 1114-1117 (2010).
34. Dickson, J. A. D. Fossil echinoderms as monitor of the Mg/Ca ratio of phanerozoic oceans. *Science* **298**, 1222-1224 (2002).
35. Ridgwell, A. A Mid Mesozoic Revolution in the regulation of ocean chemistry. *Mar Geol* **217**, 339-357 (2005).
36. Farkas, J. *et al.* Calcium isotope record of Phanerozoic oceans: Implications for

chemical evolution of seawater and its causative mechanisms. *Geochim Cosmochim Ac* **71**, 5117-5134 (2007).

37. Tarhan, L. G., Droser, M. L., Planavsky, N. J. & Johnston, D. T. Protracted development of bioturbation through the early Palaeozoic Era. *Nat Geosci* **8**, 865-869 (2015).
38. Lenton TM*, et al.* Earliest land plants created modern levels of atmospheric oxygen. *P Natl Acad Sci USA* **113**, 9704-9709 (2016).
39. Zeebe R.E. and Tyrrell T. History of carbonate ion concentration over the last 100 million years II: Revised calculations and new data. Geochim Cosmochim Ac 257, 373-392 (2019).
40. Berner, R. A. *et al.* Isotope fractionation and atmospheric oxygen: Implications for phanerozoic O-2 evolution. *Science* **287**, 1630-1633 (2000).
41. Berner, R. A., VandenBrooks, J. M. & Ward, P. D. Evolution - Oxygen and evolution. *Science* **316**, 557-558 (2007).
42. Boyle, R. A. *et al.* Stabilization of the coupled oxygen and phosphorus cycles by the evolution of bioturbation. *Nat Geosci* **7**, 671-676 (2014).
43. Kendall, B. *et al.* Uranium and molybdenum isotope evidence for an episode of widespread ocean oxygenation during the late Ediacaran Period. *Geochim Cosmochim Ac* **156**, 173-193 (2015).
44. Lowenstein, T. K., Timofeeff, M. N., Brennan, S. T., Hardie, L. A. & Demicco, R. V. Oscillations in Phanerozoic seawater chemistry: Evidence from fluid inclusions. *Science* **294**, 1086-1088 (2001).
45. Wallace, M. W. *et al.* Oxygenation history of the Neoproterozoic to early Phanerozoic and the rise of land plants. *Earth Planet Sc Lett* **466**, 12-19 (2017).
46. Mills, B. J. W., Belcher, C. M., Lenton, T. M. & Newton, R. J. A modeling case for high atmospheric oxygen concentrations during the Mesozoic and Cenozoic.

Geology **44**, 1023-1026 (2016).
47. Godderis, Y. *et al.* Onset and ending of the late Palaeozoic ice age triggered by tectonically paced rock weathering. *Nat Geosci* **10**, 382-386 (2017).
48. Berner, R. A. The long-term carbon cycle, fossil fuels and atmospheric composition. *Nature* **426**, 323-326 (2003).
49. Berner, E. K. & Berner, R. A. Global Environment: Water, Air, and Geochemical Cycles. (Princeton University Press, Upper Saddle River, NJ, ed. 2, 2012)
50. Halevy, I. & Bachan, A. The geologic history of seawater pH. *Science* **355**, 1069-1071 (2017).
51. Krom, M. D. & Berner, R. A. Adsorption of Phosphate in Anoxic Marine-Sediments. *Limnol Oceanogr* **25**, 797-806 (1980).
52. Berg, P., Rysgaard, S. & Thamdrup, B. Dynamic modeling of early diagenesis and nutrient cycling. A case study in an Arctic marine sediment. *Am J Sci* **303**, 905-955 (2003).
53. Wang, Y. F. & VanCappellen, P. A multicomponent reactive transport model of early diagenesis: Application to redox cycling in coastal marine sediments. *Geochim Cosmochim Ac* **60**, 2993-3014 (1996).
54. Reed, D. C., Slomp, C. P. & Gustafsson, B. G. Sedimentary phosphorus dynamics and the evolution of bottom-water hypoxia: A coupled benthic-pelagic model of a coastal system. *Limnol Oceanogr* **56**, 1075-1092 (2011).
55. Luff, R., Wallmann, K. & Aloisi, G. Numerical modeling of carbonate crust formation at cold vent sites: significance for fluid and methane budgets and chemosynthetic biological communities. *Earth Planet Sc Lett* **221**, 337-353 (2004).
56. Rickard, D. & Luther, G. W. Kinetics of pyrite formation by the H2S oxidation of iron(II) monosulfide in aqueous solutions between 25 and 125 degrees C: The mechanism. *Geochim Cosmochim Ac* **61**, 135-147 (1997).
57. Lenstra WK, Egger M, van Helmond NAGM, Kritzberg E, Conley DJ, Slomp CP. Large variations in iron input to an oligotrophic Baltic Sea estuary: impact on sedimentary phosphorus burial. *Biogeosciences* **15**, 6979-6996 (2018).
58. Reed DC, Gustafsson BG, Slomp CP. Shelf-to-basin iron shuttling enhances

vivianite formation in deep Baltic Sea sediments. *Earth Planet Sc Lett* **434**, 241-251 (2016).

59. Millero, F. J. Thermodynamics of the Carbon-Dioxide System in the Oceans. *Geochim Cosmochim Ac* **59**, 661-677 (1995).
60. Luo, Y. X. & Millero, F. J. Solubility of rhodochrosite (MnCO3) in NaCl solutions. *J Solution Chem* **32**, 405-416 (2003).
61. Millero, F. J. & Schreiber, D. R. Use of the Ion-Pairing Model to Estimate Activity-Coefficients of the Ionic Components of Natural-Waters. *Am J Sci* **282**, 1508-1540 (1982).
62. Lear, C.H., Elderfield, H. and Wilson, P.A., 2000. Cenozoic deep-sea temperatures and global ice volumes from Mg/Ca in benthic foraminiferal calcite. Science, 287(5451), pp.269-272.
63. Lear, C.H., Coxall, H.K., Foster, G.L., Lunt, D.J., Mawbey, E.M., Rosenthal, Y., Sosdian, S.M., Thomas, E. and Wilson, P.A., 2015. Neogene ice volume and ocean temperatures: Insights from infaunal foraminiferal Mg/Ca paleothermometry. Paleoceanography, 30(11), pp.1437-1454.
64. Van Cappellen, P. & Ingall, E. D. Benthic Phosphorus Regeneration, Net Primary Production, and Ocean Anoxia - a Model of the Coupled Marine Biogeochemical Cycles of Carbon and Phosphorus. *Paleoceanography* **9**, 677-692 (1994).
65. Van Cappellen, P. & Ingall, E. D. Redox stabilization of the atmosphere and oceans by phosphorus-limited marine productivity. *Science* **271**, 493-496 (1996).
66. Hedges, J.I. and Keil, R.G., 1995. Sedimentary organic matter preservation: an assessment and speculative synthesis. Marine chemistry, 49(2-3), pp.81-115.
67. Royer, D. L., Donnadieu, Y., Park, J., Kowalczyk, J. & Godderis, Y. Error Analysis of Co2 and O-2 Estimates from the Long-Term Geochemical Model Geocarbsulf. *Am J Sci* **314**, 1259-1283 (2014).
68. Arvidson, R. S., Mackenzie, F. T. & Guidry, M. W. Geologic history of seawater: A MAGic approach to carbon chemistry and ocean ventilation. *Chem Geol* **362**, 287-304 (2013).
69. Hansen, K. W. & Wallmann, K. Cretaceous and cenozoic evolution of seawater composition, atmospheric O-2 and CO2: A model perspctive. *Am J Sci* **303**, 94-148 (2003).
